# The Role of Selected Bacteria in Breast Cancer Initiation and Development

**DOI:** 10.3390/ijms27104585

**Published:** 2026-05-20

**Authors:** Gebremichal Gebretsadik, Syed Islam, Justin Szpendyk, Venetia Thomas, Saori Furuta

**Affiliations:** 1 MetroHealth Medical Center, School of Medicine, Case Western Reserve University, 2500 MetroHealth Drive, Cleveland, OH 44109, USA; sti12@case.edu (S.I.); jxs2143@case.edu (J.S.); vxt154@case.edu (V.T.); 2 Case Comprehensive Cancer Center, 10900 Euclid Ave., Cleveland, OH 44106, USA

**Keywords:** breast cancer, cancer initiation, *Fusobacterium nucleatum*, *Escherichia coli*, *Bacteroides fragilis*, *Staphylococcus aureus*, *Clostridium* species

## Abstract

The breast tissue microbiome is increasingly recognized as a contributor to breast cancer development. Both resident and translocated bacteria can influence carcinogenesis through several mechanisms, including chronic inflammation that promotes DNA damage, bacterial toxins with direct genotoxic effects, and microbial metabolites that alter host physiology—particularly estrogen metabolism via the “estrobolome.” Disruptions in microbial balance (dysbiosis) may further increase disease risk. Among the taxa most frequently linked to breast cancer are *Fusobacterium nucleatum*, *Escherichia coli*, *Bacteroides fragilis*, *Staphylococcus* spp., and *Clostridium* spp., each of which has been associated with distinct but sometimes overlapping roles in tumor initiation and progression. This review summarizes recent findings on these organisms and outlines the mechanisms through which they may contribute to breast carcinogenesis and metastasis. Improved understanding of host–microbe interactions in the breast could support the development of new clinical approaches, including microbial biomarkers for early detection and prognosis, as well as microbiome-targeted therapeutic strategies.

## 1. Introduction

**Figure 1:**
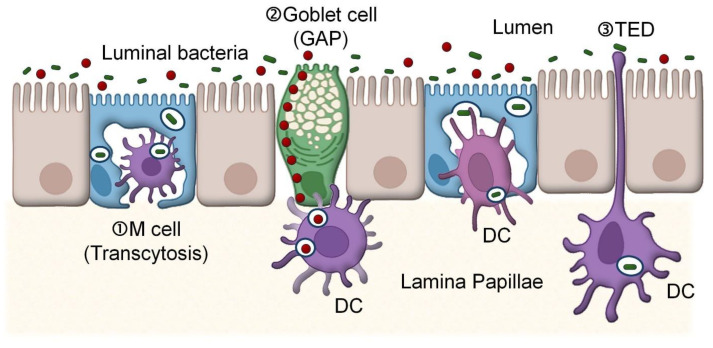
Methods of transcytosis of luminal bacteria. ① Microfold (M) cell-mediated transcytosis. ② Goblet cell-associated antigen passages (GAPs). ③ Transepithelial dendrites (TEDs) of phagocytes in the lamina propria. Created with BioRender.com. Retrieved from https://app.biorender.com/citation/69fdf07b4511a2f6c0b57710 (accessed on 18 May 2026).

Breast cancer remains a major global health challenge, with an estimated 2.3 million new cases and about 670,000 deaths each year. Improving prevention and treatment depends on a clearer understanding of its underlying biology. In recent years, attention has turned to the role of the microbiome, with several studies linking microbial imbalance (dysbiosis) in both the gut and breast tissue to increased breast cancer risk and poorer outcomes [1]. Still, whether dysbiosis directly drives cancer development or reflects disease-related changes is not yet fully resolved [2]. The relationship between microbes and cancer appears to be multifaceted, involving interactions among diverse microbial communities and the host [3,4,5,6]. One proposed mechanism is the so-called ‘gut–breast axis’, which was initially conceived in relation to pregnancy and lactation under the heightened influence of female sexual hormones [7,8,9,10,11,12]. Low levels of gut to breast bacterial translocation appear to take place during pregnancy to increase the abundance of beneficial gut bacteria, such as *Bifidobacterium* and *Lactobacillus*, in breast tissues and milk [8,9,13,14]. This allows maternal gut bacteria to be transferred to infants’ guts through breast milk [15]. Although still largely hypothetical, the gut–breast axis may extend beyond pregnancy and lactation, with potential long-term or even pregnancy-independent interactions between gut-derived microbes, immune cells, and the mammary microenvironment [9,16]. In the gut-breast axis model, disruption of the intestinal barrier allows gut bacteria or their metabolites to enter circulation and reach distant tissues, including the mammary gland [17]. Immune cells such as dendritic cells and macrophages are proposed to transport internalized bacteria from the gut to the breast and milk (Figure 1) [18,19,20]. On the other hand, the opportunistic oral pathogen *Fusobacterium nucleatum* has been shown to disseminate primarily via the bloodstream to tumor sites [21]. In parallel, circulating microbial metabolites can influence breast tissue without direct bacterial colonization. While these observations support a connection between gut microbes and breast cancer, the key pathways and mediators involved remain to be fully defined [17].

Recent studies show that breast tumors contain distinct microbial communities that may play a role in cancer development and treatment response [6]. These tumor-associated microbes can influence carcinogenesis through several overlapping mechanisms. Bacterial components can activate innate immune receptors, driving inflammatory signaling that contributes to DNA damage and tissue remodeling. Some bacteria and their metabolites can also directly promote genomic instability and activate oncogenic pathways such as MAPK, PI3K/AKT, and NF-κB, supporting tumor cell proliferation, survival, and migration. In addition, microbes within the tumor environment may dampen anti-tumor immune responses and help establish an immunosuppressive niche. Their metabolic products can further affect host physiology, including estrogen metabolism, oxidative stress, and nutrient availability, all of which can support tumor growth [6].

A number of bacterial taxa have been repeatedly linked to breast cancer [2,6,22,23,24]. Among them, *Fusobacterium nucleatum*, *Escherichia coli*, *Staphylococcus* spp., *Bacteroides fragilis*, and *Clostridium* spp. are among those most consistently reported. These organisms have been detected in breast tumors and are associated with processes such as chronic inflammation, DNA damage, immune modulation, and metabolic changes. For example, *F. nucleatum* has been linked to immune evasion and disease progression [21], while certain *E. coli* strains produce genotoxins that can damage DNA [25]. *B. fragilis* and *Clostridium* species are known to contribute to pro-inflammatory signaling and the production of metabolites that may promote tumor development [1]. Members of the *Staphylococcus* genus are also commonly found in breast tissue and may influence local immune responses [26]. Given their frequent association with breast cancer, these five groups are used here as representative examples for further discussion.

## 2. Major Bacterial Contributors to Breast Cancer Development

### 2.1. Fusobacterium nucleatum in Breast Cancer

#### 2.1.1. Overview of *Fusobacterium nucleatum*

Clinical studies have repeatedly found that *Fusobacterium nucleatum* (*Fn*) is enriched in breast tumors and is linked to disease progression. Analyses of patient samples show higher levels of *Fn* DNA in tumors compared to adjacent normal tissue, particularly in tumors with elevated expression of Gal-GalNAc glycans that facilitate bacterial adhesion [21,27]. Higher intratumoral *Fn* levels have also been associated with larger tumors and increased metastasis, suggesting a connection with more aggressive disease [21,27]. Spatial and molecular studies indicate that *Fn* can localize within both tumor cells and the surrounding microenvironment, pointing to direct interactions with host tissues that may influence tumor behavior [28]. There is also evidence that *Fn* may originate from outside the breast, with proposed translocation from the oral cavity or gut via the bloodstream, in line with the gut–breast axis hypothesis [17,21]. Clinically, *Fn*-positive tumors tend to show increased inflammatory signaling alongside reduced infiltration of cytotoxic CD8^+^ T cells, consistent with a role in immune suppression and tumor immune evasion [3,29]. In addition, epidemiological studies have linked higher oral levels of *Fn*—such as in periodontitis—with increased breast cancer risk [27]. Together, these findings support a role for *Fn* as both a marker of disease progression and a potential contributor to tumor aggressiveness.

#### 2.1.2. General Characteristics of *Fusobacterium nucleatum*

*Fn* is a Gram-negative, obligate anaerobic bacterium commonly found in the human oral microbiota, where it behaves as both a commensal organism and an opportunistic pathogen. It is best known for its role in anaerobic subgingival plaque but can also colonize sites beyond the oral cavity [3,21,30]. *Fn* prefers nutrient-rich, low-oxygen environments and relies on several virulence factors—especially adhesion proteins—to attach to host cells and evade immune responses (Table 1) [30]. Taxonomically, *Fn* belongs to the family *Fusobacteriaceae* within the phylum *Fusobacteriota*. Although it has traditionally been grouped as a single species, it includes four recognized subspecies—*F. nucleatum* subsp. *nucleatum*, *animalis*, *polymorphum*, and *vincentii*—which genomic studies increasingly suggest may represent distinct species [30,31]. Morphologically, *Fn* is characterized by elongated, non-motile, non-spore-forming rods with tapered ends [30]. Host interactions of *F. nucleatum* are mediated through highly specific adhesin–receptor contacts. Binding of Fap2 to host glycans and FadA to E-cadherin depends not only on primary sequence motifs but also on structural features such as oligomerization, conformational flexibility, and local receptor availability, all of which influence binding avidity and downstream effects including β-catenin signaling [32,33,34]. There is also growing evidence that differences among *F. nucleatum* subspecies can alter these adhesins at the sequence level, with potential consequences for binding strength, receptor preference, and tissue distribution [32,35,36]. Such variability may help explain strain-specific differences in immune modulation and pathogenic behavior. At the same time, the lack of detailed structural and thermodynamic data continues to limit a full mechanistic understanding of these interactions. Filling these gaps will be important for developing strategies that target adhesin–receptor binding, either by directly blocking interaction interfaces or by destabilizing key structural states required for binding [32,36,37].

#### 2.1.3. Breast Tumor-Association of *Fusobacterium nucleatum*

*Fn* has been repeatedly detected at higher levels in breast tumor tissues, including both formalin-fixed paraffin-embedded (FFPE) samples and fresh specimens [3,4,21,27,28,52,53]. Multiple studies report increased *Fn* DNA in tumors compared to adjacent normal breast tissue. Its presence is particularly notable in tumors with high expression of the tumor-associated glycan Gal-GalNAc, suggesting some degree of selective colonization. Higher *Fn* levels have also been linked to larger tumor size and greater metastatic potential [21,27,54]. Epidemiological studies connecting periodontal disease with breast cancer further point to a possible role for this oral bacterium in disease development [55]. *Fn* may reach breast tissue through several routes. Hematogenous spread is considered a key pathway, supported by evidence that the bacterial adhesin Fap2 can recognize Gal-GalNAc on tumor cells [21]. Other routes have also been proposed, including direct transfer from the oral cavity via nipple–areolar contact during breastfeeding or sexual activity, which could allow microbial entry into breast tissue [53].

#### 2.1.4. Pro-Tumor Functions of *Fusobacterium nucleatum*

**Figure 2:**
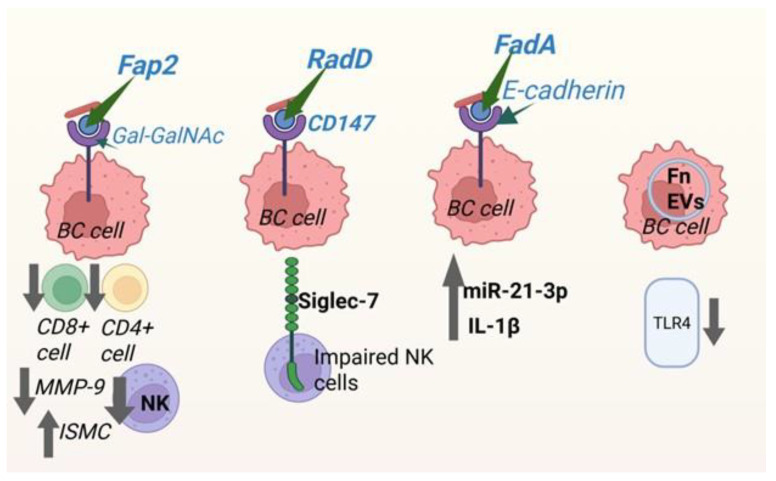
Virulent factors of *F. nucleatum* and immune modulation in breast cancer progression. Different *Fn* adhesins—Fap2, RadD, and FadA—mediate interactions with breast cancer cells via specific host receptors such as Gal-GalNAc, CD147, and E-cadherin, respectively, promoting pro-tumor signaling. These interactions contribute to immune evasion and tumor progression by suppressing CD8^+^ and CD4^+^ T cell activity, impairing NK cell function via Siglec-7 signaling, and increasing pro-inflammatory mediators such as IL-1β and miR-21-3p. Additionally, bacterial extracellular vehicles (EVs) influence Toll-like receptor 4 (TLR4) signaling. Upward or downward arrows indicate up- or down-regulation of signals, respectively. Created with BioRender.com. Retrieved from https://app.biorender.com/citation/69fdf114d8bf502e7bc50070 (accessed on 18 May 2026).

Experimental studies show that *Fn* can promote mammary tumor growth and increase metastatic spread in vivo [21]. Exposure to *Fn* has also been shown to trigger inflammatory responses, alter chemokine expression, and induce broad epigenomic changes in host cells, with the bacterium often detected within tumor cells themselves [28,56]. Many of these effects are linked to *Fn*’s virulence-associated proteins, sometimes referred to as the “FusoSecretome.” Key factors such as Fusobacterium autotransporter protein 2 (Fap2), Fusobacterium adhesin A (FadA), and RadD play central roles in adhesion, colonization, immune evasion, and activation of oncogenic signaling pathways (Figure 2, Table 1) [30,39,57]. These adhesins also contribute to biofilm formation, which can support bacterial persistence in the tumor environment. In addition, *Fn* has been shown to influence host gene and protein expression, including upregulation of VEGFD and PAK1, both of which are linked to increased cell proliferation, migration, and invasion [28].

Fap2, a galactose-sensitive lectin, plays an important role in how *Fn* targets tumor cells by binding to Gal-GalNAc structures that are often overexpressed on breast cancer cells. Strains that express Fap2 show stronger attachment to tumor cells than Fap2-deficient mutants, supporting its involvement in tumor colonization and possibly metastasis [21]. Beyond adhesion, Fap2 can also dampen anti-tumor immunity by interacting with inhibitory receptors such as TIGIT on natural killer (NK) cells and CEACAM1 on T cells, contributing to immune evasion [30].

*Fn* also influences several oncogenic signaling pathways. For example, FadA has been shown to activate the Wnt/β-catenin pathway, which is linked to tumor development [38]. Exposure to *Fn* can promote epithelial–mesenchymal transition (EMT) through pathways including Wnt, NF-κB, and IL-17, while disrupting cell–cell adhesion structures such as tight junctions and focal adhesions [4,58]. These changes are associated with increased invasiveness and metastatic potential. Inflammatory signaling is another key component. *Fn* can stimulate IL-1β production through activation of the NLRP3 inflammasome, and blocking this pathway has been shown to reduce *Fn*-driven tumor growth [59]. It may also enhance cancer cell migration through the miR-21-3p/FOXO3 axis, where increased miR-21-3p suppresses FOXO3 and promotes motility [57].

In the tumor microenvironment, *Fn* appears to shift immune responses in ways that favor tumor progression. Studies in vivo report reduced infiltration of CD4^+^ and CD8^+^ T cells in *Fn*-associated tumors, along with increased expression of inflammatory and immunosuppressive factors such as MMP-9 and various chemokines [21]. *Fn* can also promote immune escape through activation of the NF-κB/PD-L1 pathway, which reduces CD8^+^ T-cell activity [3]. Other mechanisms include recruitment of myeloid-derived suppressor cells (MDSCs) and increased expression of immune checkpoint molecules such as PD-L1 and CD47 [2]. Direct interactions with immune cells also play a role: RadD binding to Siglec-7 can inhibit NK cell function, while Fap2 interaction with TIGIT suppresses both NK cells and tumor-infiltrating T cells [39,60]. Together, these effects contribute to a more immunosuppressive tumor environment and may reduce responsiveness to immunotherapy [29].

*Fn*-derived extracellular vesicles (EVs) add another layer of influence by activating Toll-like receptor 4 (TLR4), which has been linked to increased proliferation, migration, and invasion of breast cancer cells; these effects are reduced when TLR4 is inhibited [27]. Higher levels of *Fn* DNA in breast tissue have also been associated with tumor progression and metastasis [27]. In addition, *Fn* may contribute to metabolic changes within tumors. Its metabolites—including succinate, formate, ADP-heptose, and butyrate—have been linked to inflammation, immune modulation, and therapeutic resistance [30]. Activation of TLR4 by bacterial lipopolysaccharide (LPS) can further drive NF-κB signaling and increase expression of anti-apoptotic proteins such as Bcl-2 and Bcl-xL, supporting tumor cell survival [30]. There is also evidence that *Fn* may interact with pathways like MAPK and Ras, promoting proliferation and migration [2,28]. Moreover, in both normal mammary tissue and breast cancer mouse model, *Fn* induces inflammation, DNA damage, and abnormal cellular proliferation, contributing to the development of precancerous metaplastic lesions [61]. Taken together, findings from both in vitro and in vivo studies support a model in which *Fn* not only colonizes breast tumors but also actively contributes to tumor growth, metastasis, and immune evasion through a combination of inflammatory, signaling, and metabolic effects. These observations point to *Fn* as a potentially important microbial factor in breast cancer progression and suggest that targeting tumor-associated microbes, or modulating the microbiome more broadly, could be a useful direction for future therapeutic strategies.

### 2.2. Escherichia coli in Breast Cancer

#### 2.2.1. Overview of *Escherichia coli*

Clinical and translational studies indicate that *Escherichia coli* (*E. coli*) is present and functionally relevant in breast cancer–associated microbiota. Analyses of human breast tissues have detected *E. coli* in tumor-adjacent and cancerous samples, often with greater abundance compared to healthy controls, suggesting its association with disease states [62]. Notably, isolates obtained from breast cancer patients demonstrate the ability to induce DNA double-strand breaks in host cells, highlighting their genotoxic potential and supporting a direct role in genomic instability [62]. A key clinical feature of pathogenic *E. coli* strains is the presence of the *pks* genomic island, which encodes the genotoxin colibactin; this compound has been implicated in DNA damage and carcinogenesis within breast tissue [62]. In addition, recent metagenomic analyses of patient microbiomes have identified *E. coli* and its metabolites—particularly siderophores involved in iron acquisition—as significantly associated with breast cancer, with potential roles in promoting tumor growth and angiogenesis [63]. Beyond tissue-level observations, experimental findings using clinically relevant models demonstrate that pathogenic *E. coli* can induce oxidative stress and DNA damage via activation of enzymes such as spermine oxidase (SMOX), linking microbial presence to inflammation-driven tumor progression [64]. Collectively, these clinical findings support a model in which *E. coli*, particularly pathogenic and *pks*-positive strains, contributes to breast carcinogenesis through genotoxicity, metabolic adaptation, and modulation of the tumor microenvironment.

#### 2.2.2. General Characteristics of *Escherichia coli*

*E. coli* is a Gram-negative, facultative anaerobic rod that normally resides in the lower intestine, making up about 0.1% of the gut microbiota [65,66]. Despite this relatively low abundance, it plays an important role in gut function. As one of the first colonizers of the neonatal intestine, it contributes to digestion, nutrient production, and the establishment of conditions that support obligate anaerobes [67]. Most strains are harmless or beneficial, involved in vitamin K2 synthesis and the production of colicins that inhibit pathogens such as *Salmonella* [68,69,70,71]. Its metabolic flexibility also allows it to survive outside the host for several days [72,73], and it remains a widely used model organism in microbiology. At the same time, *E. coli* is highly diverse, with more than 700 serotypes identified [74]. While many strains are commensal, pathogenic variants such as enteropathogenic (EPEC) and enterotoxigenic (ETEC) *E. coli* can cause gastrointestinal infections and are typically transmitted via the fecal–oral route [75].

Phylogenetically, *Escherichia coli* is divided into six major groups (A, B1, B2, D, E, and *Shigella*). Commensal strains are most commonly found in phylogroup A, which predominates in healthy individuals and environmental reservoirs such as soil and water [76]. These strains can also influence chemotherapy responses by altering drug activity, sometimes increasing toxicity or reducing efficacy of agents such as gemcitabine, doxorubicin, and mitoxantrone [77]. In contrast, phylogroup B2 includes strains that frequently carry virulence genes (Table 1) and are strongly associated with extraintestinal infections, including urinary tract infections, meningitis, and septicemia [78,79]. A subset of B2 strains harbor the *pks* genomic island encoding colibactin, which has been implicated in carcinogenesis, particularly colorectal cancer, and is increasingly considered in breast cancer contexts [80]. B2 strains also show enhanced biofilm formation, virulence, and antibiotic resistance [81]. These strains are classified as extraintestinal pathogenic *E. coli* (ExPEC) and include globally disseminated lineages such as ST131, which commonly carry ESBL genes (e.g., CTX-M) and fluoroquinolone resistance. Their virulence is supported by adhesins, toxins, and iron acquisition systems that facilitate invasive disease. In cancer patients, repeated antibiotic exposure can select for B2 strains, contributing to dysbiosis, infection risk, and altered treatment outcomes [82,83,84]. B2 strains have also been detected at higher abundance in breast tumor tissue compared to normal tissue [62,78,79], potentially reflecting adaptation to the tumor microenvironment. Their pathogenicity is shaped by horizontally acquired virulence determinants maintained within specific lineages [85].

#### 2.2.3. Breast Tumor-Association of *Escherichia coli*

Studies of human breast tissue have found that *E. coli* is detected more often in malignant samples than in benign or healthy breast tissue [62]. Some isolates from breast tumors also appear to be functionally relevant, as they can induce DNA damage in epithelial cells, particularly through the *pks* island and its genotoxin colibactin. This links the presence of certain *E. coli* strains with genomic instability and potential contributions to carcinogenesis [62,86]. In addition, the tumor microenvironment itself—especially conditions such as hypoxia and altered nutrient availability—may favor the survival of facultative anaerobes like *E. coli*, helping explain their enrichment in tumor-associated niches.

The origin of *E. coli* in breast tissue is still not fully clear. The prevailing view is that these bacteria originate from the gut microbiota and reach the mammary gland through hematogenous or lymphatic spread [87]. Several routes have been proposed for crossing the intestinal barrier, including uptake via microfold (M) cells, sampling by dendritic cell extensions, and goblet cell–associated antigen passages (GAPs) (Figure 1) [28,88]. GAP-mediated translocation, which is regulated by acetylcholine and inhibited by epidermal growth factor signaling, has been suggested as an important pathway for dissemination of pathogenic *E. coli* strains [88,89]. Some strains, often referred to as translocating *E. coli* (TEC), seem particularly adept at crossing barriers and altering host environments. For example, adherent-invasive *E. coli* (AIEC) strain HMLN-1, isolated from mesenteric lymph nodes, has been linked to inflammatory conditions and cancer [90,91,92]. In addition to gut-derived spread, local entry routes have also been proposed. During conditions such as mastitis or breast abscesses, *E. coli* may enter mammary ducts directly and become part of the local breast microbiota [93].

#### 2.2.4. Pro-Tumor Functions of *Escherichia coli*

Within breast tissue, *E. coli* may contribute to carcinogenesis mainly through genotoxic and inflammatory effects. Strains carrying the *pks* genomic island produce colibactin, a genotoxin that causes DNA interstrand crosslinks and double-strand breaks, resulting in genomic instability and mutation accumulation (Table 1) [25,94]. These strains can also kill normal cells or drive them into senescence, which may indirectly support tumor growth by reshaping the surrounding environment and releasing factors that favor nearby cancer cells [28,41,42]. There is also evidence that colibactin exposure can contribute to chemotherapy resistance. Tumors exposed to *pks*-positive strains often show increased lipid droplet formation, which provides an energy source for cancer cells and can impair immune cell activity [95]. At the same time, activation of DNA damage response pathways such as homologous recombination may allow tumor cells to better tolerate DNA-damaging therapies [96]. In some cases, *pks*-positive *E. coli* has been detected within tumor cells and macrophages, where it may further drive inflammation through mediators like COX-2 [97].

*E. coli* can also influence tumor biology through secreted metabolites. Exposure to bacterial secretomes has been shown to promote epithelial–mesenchymal transition (EMT) in mammary epithelial cells and to stimulate fibroblasts to release pro-inflammatory and pro-tumorigenic cytokines, including HGF, IL-1α, IL-4, FGF-4, RANTES, and CXCL5 [43]. Metabolomic profiling has also identified several *E. coli*–derived compounds associated with breast cancer progression, such as N-acetyl-L-methionine, nicotinamide riboside, N-acetylneuraminic acid, mannose-1-phosphate, and glutathionylspermidine [44]. These metabolites are involved in pathways related to carbohydrate and amino acid metabolism, lipid signaling, and nucleotide synthesis, all of which can support tumor growth, survival, and treatment resistance [44].

#### 2.2.5. Anti-Tumor Functions of Escherichia coli

Despite its association with tumorigenesis, *E. coli* is also being investigated as a therapeutic platform. Progress in synthetic biology has made it possible to engineer non-pathogenic *E. coli* strains that can preferentially localize to tumors and act as delivery systems for therapeutic molecules [98]. One well-studied example is the probiotic strain *E. coli* Nissle 1917 (EcN), originally isolated by Alfred Nissle. EcN naturally produces microcins that inhibit competing pathogenic bacteria [99]. More recently, engineered EcN strains have been adapted to deliver immunotherapeutic payloads and enhance anti-tumor immune responses, particularly in colorectal cancer models [100]. These engineered bacterial systems are now being evaluated in early-stage clinical studies as potential tools for targeted cancer therapy [100,101].

### 2.3. Bacteroides fragilis in Breast Cancer

#### 2.3.1. Overview of *Bacteroides fragilis*

Clinical evidence linking *Bacteroides fragilis* to breast cancer is still relatively limited compared with some other microbial taxa; however, emerging data suggest it may be present in the breast tumor microenvironment and potentially biologically relevant. Microbiome studies of human breast tissues have detected members of the *Bacteroides* genus, including *B. fragilis*, in both tumor and adjacent normal tissues, indicating that gut-associated anaerobes can reach and persist in the breast environment and may contribute to local dysbiosis [102,103]. Experimental work provides further support for a possible role. In particular, enterotoxigenic *B. fragilis* (ETBF) has been shown in relevant models to translocate from the gut to mammary tissue, where its toxin (BFT) can be detected and is associated with ductal hyperplasia, stromal inflammation, and increased immune cell infiltration—changes that are often linked to early tumor-promoting processes (Table 1) [45]. Evidence from other epithelial cancers also helps solidify these findings. ETBF-driven inflammation is characterized by elevated cytokines such as IL-6 and IL-8, along with increased reactive oxygen species (ROS), all of which are known to contribute to tumor development and are also relevant in breast cancer biology [104,105]. Although large clinical datasets quantifying B. fragilis specifically in breast tumors are still lacking, its repeated detection in breast cancer–associated microbiomes supports a potential role in disease. Mechanistic and in vivo studies further show that ETBF can drive inflammatory changes. Together, these findings support a model in which B. fragilis may contribute to breast carcinogenesis through toxin-mediated epithelial disruption, inflammation, and gut–breast microbial interactions.

#### 2.3.2. General Characteristics of *Bacteroides fragilis*

In humans, the colon contains the largest population of anaerobic bacteria, with *Bacteroides* species making up roughly 25% of the community [106]. Within the gut microbiome, *Bacteroides* is one of the dominant genera and remains a key component at the phylum level as well [107]. Although the genus includes a number of species, *Bacteroides fragilis* (*B. fragilis*) is the most commonly implicated pathogenic member [106]. It is an anaerobic, Gram-negative, rod-shaped bacterium and typically accounts for about 1–14% of *Bacteroides* found in human feces [108]. Colonization likely begins early in life, probably during birth, and in most individuals *B. fragilis* persists as a largely mutualistic commensal [23]. Longitudinal studies support its high prevalence in healthy populations; for example, it has been detected in up to 87% of fecal samples from healthy individuals and is often the dominant *Bacteroides* species in these cohorts [109]. Despite this generally commensal role, some strains can be pathogenic. Enterotoxigenic *B. fragilis* (ETBF) produces *B. fragilis* toxin (BFT), a heat-labile metalloprotease that has been linked to diarrheal disease and chronic inflammatory conditions [110,111,112].

#### 2.3.3. Breast–Tumor Association of *Bacteroides fragilis*

While *B. fragilis* has been studied most extensively in colorectal cancer, there is growing evidence that it may also play a role in breast carcinogenesis. Microbiome analyses of human breast tissues have detected members of the *Bacteroides* genus in both tumor and adjacent samples, suggesting that gut-associated anaerobes can reach and persist within the breast tumor microenvironment [62,102,103]. Direct evidence for *B. fragilis* in breast cancer is still limited; however, its well-described pro-inflammatory and tumor-promoting effects in other epithelial cancers make it a plausible contributor in breast cancer biology as well [104,113]. Experimental data further support this possibility. In a study by Parida et al., oral administration of enterotoxigenic *B. fragilis* (ETBF) in mice led to the detection of *B. fragilis* toxin (BFT) in breast tissue within five days. This was accompanied by clear histological changes, including enlargement of terminal end buds, thickening of ductal epithelium, stromal infiltration, collagen deposition, epithelial hyperplasia, and increased T-cell infiltration. Together, these findings suggest that *B. fragilis* can translocate from the gut to the mammary gland and induce tissue-level changes consistent with early tumor-promoting processes [45].

#### 2.3.4. Pro-Tumor Functions of *Bacteroides fragilis*

Chronic inflammation is widely recognized as a driver of carcinogenesis and is estimated to contribute to about 20% of epithelial cancers [64,114]. Enterotoxigenic *B. fragilis* (ETBF) has been shown to promote both inflammation and oxidative stress. In chronic inflammatory states, dysregulated immune signaling and elevated reactive oxygen species (ROS) activate pro-inflammatory cytokines and oncogenic pathways, eventually leading to DNA damage, tumor initiation, and progression [64]. One important mechanism involves spermine oxidase (SMO), a polyamine catabolic enzyme that converts spermine to spermidine while generating hydrogen peroxide (H_2_O_2_), a major source of ROS [64,115]. B. fragilis toxin (BFT) can upregulate SMO in epithelial cells, increasing ROS levels and promoting oxidative DNA damage, thereby contributing to carcinogenic processes [64]. Sustained ROS production also activates stress-related pathways such as ERK/p38 MAPK, which in turn stimulates nuclear factor erythroid 2–related factor 2 (Nrf2) and increases expression of heme oxygenase-1 (HO-1) [103]. BFT-induced HO-1 expression has been associated with delayed apoptosis, allowing damaged cells to survive longer and maintain inflammatory signaling, which together support a pro-tumor environment [116]. While these mechanisms have been mainly studied in intestinal epithelium, oxidative stress and chronic inflammation are broadly relevant across epithelial tissues, including the breast. In breast tumors, hypoxia further enhances ROS production and promotes recruitment of immunosuppressive cells and cytokines, supporting tumor growth and survival [46]. This suggests that ROS-driven inflammation may represent a shared mechanism linking microbial dysbiosis with cancer progression across different tissues.

ETBF also promotes tumorigenesis through cytokine induction. Interleukin-8 (IL-8), a key chemokine involved in neutrophil recruitment, is frequently upregulated in breast cancer and contributes to tumor progression by enhancing survival, invasion, and angiogenesis [47,117]. BFT induces IL-8 expression through AP-1 and MAPK signaling pathways in epithelial cells [118,119]. It also promotes IL-8 production indirectly by disrupting epithelial integrity: BFT triggers cleavage of E-cadherin via upregulation of matrix metalloproteinase-7 (MMP-7), weakening cell–cell adhesion [120,121,122,123]. Loss of E-cadherin releases β-catenin, which activates NF-κB, MAPK, and STAT3 signaling, further increasing IL-8 production, ROS generation, and epithelial permeability [122,123]. Increased permeability may, in turn, facilitate bacterial translocation into circulation and potentially support dissemination to distant sites such as the breast [124].

Interleukin-6 (IL-6) is another central cytokine in ETBF-associated inflammation. Although it has normal roles in immune regulation, sustained IL-6 signaling promotes tumor cell proliferation, immune evasion, and survival [121]. It acts mainly through the gp130/JAK/STAT3 pathway, which drives oncogenic transcription programs [125,126]. IL-6 expression can also increase following E-cadherin disruption [121]. While most evidence comes from colorectal cancer models, the IL-6/JAK/STAT3 axis is already well established in breast cancer progression, suggesting a possible overlap between ETBF-driven inflammation and breast tumor biology [117,125,126], even if direct evidence in breast tissue is still limited. IL-17 signaling adds another layer to this inflammatory network. In breast cancer, IL-17A and IL-17B are the most relevant forms. IL-17A, produced mainly by Th17 cells, promotes tumor progression directly by acting on IL-17 receptors on tumor cells, inducing IL-6 and CCL20 and creating a feedback loop that sustains inflammation [127,128]. It can also act indirectly by promoting neutrophil expansion and polarization toward tumor-promoting phenotypes, contributing to metastasis [127,128]. IL-17B signals through IL-17RB and activates NF-κB and ERK1/2 pathways, promoting proliferation, migration, and invasion while inhibiting apoptosis [127]. Overall, these findings suggest that ETBF, and *B. fragilis* more broadly, can drive cancer-associated inflammation through ROS generation, epithelial barrier disruption, and sustained cytokine signaling. While much of the mechanistic evidence comes from colorectal models, the same pathways are highly relevant in breast cancer biology. However, direct experimental confirmation in breast tissue is still limited and remains an important area for further study.

### 2.4. Staphylococcus aureus in Breast Cancer

#### 2.4.1. Overview of *Staphylococcus aureus*

Clinical studies of the breast cancer-associated microbiota have identified *Staphylococcus aureus* (*S. aureus*) as a frequent but context-dependent member of breast tissue communities. Sequencing data from human samples indicate that *Staphylococcus* species are present in both healthy and diseased breast tissues, although their abundance varies with disease state. In general, *Staphylococcus*, including *S. aureus*, is more commonly found in normal and benign breast tissue, while reduced levels are often reported in malignant tumors [62,102]. Breast cancer-associated dysbiosis also involves shifts in *Staphylococcus* representation compared with adjacent normal tissue [129]. Even when less abundant in tumors, *S. aureus* may still be relevant because of its interactions with the tumor microenvironment. It can affect cancer progression indirectly through immune modulation; for instance, *S. aureus*-induced inflammation can drive neutrophil recruitment and the formation of neutrophil extracellular traps (NETs), which may capture circulating tumor cells and support metastasis [48]. On the other hand, *S. aureus*-derived extracellular vesicles (EVs) have been shown to influence signaling in breast cancer cells, including inhibition of PI3K/AKT and ERK pathways in the presence of tamoxifen, which may enhance treatment sensitivity [130]. Clinically, *S. aureus* is also a well-known cause of mastitis and breast abscesses. These inflammatory conditions can alter the local tissue environment and, over time, may contribute to changes in cancer risk or progression through persistent inflammation [131]. In some studies, *S. aureus* has also been found alongside viral infections such as human papillomavirus (HPV) in breast lesions, where combined microbial–viral interactions have been linked to genetic changes including MED12 mutations [132]. Overall, *S. aureus* appears to be a stable but context-dependent component of the breast microbiome. While it is often more abundant in normal or benign tissue, its ability to shape immune responses, drive inflammation, and influence therapeutic pathways suggests it may still play an important role in breast cancer biology.

#### 2.4.2. General Characteristics of *Staphylococcus aureus*

*S. aureus* is a Gram-positive, facultative anaerobic bacterium best known for its clinical relevance and ability to adapt to a wide range of environments [26,133,134]. It behaves both as a commensal organism and an opportunistic pathogen, colonizing human skin and mucosal surfaces early in life. Common colonization sites include the nasal cavity, oropharynx, axilla, perineum, and gastrointestinal tract. Outside the host, it is also found in environmental reservoirs such as air, soil, water, and food [135,136]. Although often carried without symptoms, *S. aureus* can cause infections ranging from mild skin lesions to severe, life-threatening systemic disease, particularly in immunocompromised individuals. Its pathogenicity is linked to a broad set of virulence factors that support adhesion, tissue invasion, and immune modulation (Table 1). For colonization, fibronectin-binding proteins allow attachment to host extracellular matrix components such as fibronectin, elastin, and plasminogen [137,138]. Once established, the bacterium can invade tissues using enzymes like hyaluronidase and collagenase, which break down extracellular matrix barriers. It also produces a range of toxins. Panton–Valentine leukocidin (PVL) targets and destroys neutrophils, while α-hemolysin damages epithelial, endothelial, and blood cells [136,139,140,141,142]. In addition, *S. aureus* can strongly disrupt immune regulation through superantigens, which trigger broad activation of T and B cells without the usual antigen specificity. Staphylococcal protein A (SpA) acts as a B-cell superantigen, while staphylococcal enterotoxins and toxic shock syndrome toxin-1 (TSST-1) drive excessive T-cell activation and cytokine release, often leading to marked immune dysregulation [142]. To further complicate the situation, antibiotic-resistant *S. aureus*, particularly methicillin-resistant *S. aureus* (MRSA), is a major clinical concern in immunocompromised populations, including cancer patients. MRSA strains carry the *mecA* gene encoding an altered penicillin-binding protein (PBP2a), which confers resistance to all β-lactam antibiotics. Many hospital-associated and community-associated MRSA lineages also exhibit multidrug resistance and enhanced virulence through toxins, adhesins, and immune evasion factors. In oncology settings, these strains are associated with bloodstream infections, surgical site infections, and pneumonia, and their persistence is facilitated by selective pressure from repeated antibiotic exposure [143,144,145].

#### 2.4.3. Breast–Tumor Association of *Staphylococcus aureus*

As a member of the human microbiota, *S. aureus* is present at multiple body sites, including the skin, nasal cavity, gut, vagina, and breast tissue, and has also been detected in breast milk [134]. Its ability to tolerate different pH environments, form biofilms, and evade host immune responses supports long-term persistence in many individuals. In the context of breast cancer, its role appears to depend on the tissue setting. Several studies have reported higher levels of *S. aureus* in normal breast tissue compared to malignant samples, with intermediate or sometimes increased abundance in benign lesions such as fibroadenomas [5,24,146,147]. This pattern suggests its closer association with tissue homeostasis or early remodeling rather than its direct role in tumor initiation. Observations from clinical breast tumor specimens are consistent with this view. Although *S. aureus* tends to be less abundant in malignant tissue, *Staphylococcus* species can still be detected in tumor-associated areas, including stromal and peri-ductal regions, where they have been linked to local inflammatory and immune features [5,24,146,147]. These findings may reflect interactions with infiltrating immune cells or changes in epithelial integrity, and in some cases could involve biofilm formation that affects local tissue organization. More broadly, shifts in microbial composition across normal, benign, and malignant breast tissues suggest that these communities change with disease progression rather than acting as fixed drivers [62,148]. Taken together, the available evidence points to a context-dependent role for *S. aureus*. While it is unlikely to function as a primary oncogenic agent, it may influence the local microenvironment—particularly in earlier or non-malignant states—through effects on immune responses and tissue structure during cancer progression.

#### 2.4.4. Pro-Tumor Functions of *Staphylococcus aureus*

*S. aureus* can shape the tumor microenvironment (TME) in breast cancer both through its direct presence in tissues and indirectly by altering host immune and inflammatory responses [137,149,150]. One proposed mechanism involves neutrophil modulation. In a murine model, nasal colonization with *S. aureus* led to increased neutrophil recruitment and formation of neutrophil extracellular traps (NETs). While NETs are part of the innate immune response and help trap pathogens, they can also create a pro-inflammatory environment that supports tumor progression and facilitate the capture of circulating tumor cells, promoting metastasis [48,151]. At the same time, neutrophils can suppress cytotoxic T-cell function, further weakening anti-tumor immunity and contributing to an immunosuppressive TME [48,152]. There is also evidence that *S. aureus* may interact with other oncogenic factors. Co-detection with human papillomavirus (HPV) has been linked to alterations in the MED12 gene, which regulates transcriptional activity. Disruption of MED12 has been associated with activation of GLI3-dependent Sonic Hedgehog (SHH) signaling, leading to increased proliferation and colony formation in breast cancer cells [5,153,154]. Experimental studies in triple-negative breast cancer (TNBC) models suggest additional complexity. *S. aureus* has been shown to localize within the cytoplasm of breast cancer cells, with bacterial load varying between different cell lines [49]. Infection produced variable effects on cell viability, with some TNBC subtypes showing reduced growth and others appearing more resistant, highlighting heterogeneity in host–pathogen responses. At the molecular level, *S. aureus* can influence immune checkpoint pathways. While infection alone does not significantly change PD-L1 expression, combined stimulation with interferon-γ (IFN-γ) increases PD-L1 levels in certain TNBC cell lines through activation of the JAK2/STAT1 pathway [49]. Increased STAT1 phosphorylation suggests that *S. aureus* may enhance IFN-γ–driven immune evasion. In addition, it can upregulate Toll-like receptor 2 (TLR2), and activation of this pathway has been associated with tumor-promoting inflammatory signaling and cancer cell survival [155,156].

#### 2.4.5. Anti-Tumor Functions of *Staphylococcus aureus*

*S. aureus* may also have context-dependent effects that are not strictly pro-tumorigenic. In particular, extracellular vesicles (EVs) derived from *S. aureus* have been shown to influence signaling pathways in breast cancer cells. In estrogen receptor–positive (ER^+^) cell lines such as MCF-7 and BT-474, exposure to these EVs increased the cytotoxic effect of tamoxifen [157]. This was accompanied by reduced activity of survival pathways, including PI3K/AKT and ERK signaling. These observations suggest that bacterial EVs can modulate estrogen-related signaling, potentially linking them to microbial factors involved in estrogen metabolism within the broader “estrobolome” framework [158,159,160]. At the same time, *S. aureus* can produce enzymes such as β-glucuronidase that deconjugate estrogens, potentially increasing levels of bioactive estrogen and, in other contexts, supporting hormone-driven tumor growth. The exact components of *S. aureus* EVs responsible for these effects are still not well defined, although extracellular adherence proteins have been proposed as a possible contributor to immune and signaling modulation [161]. Taken together, the role of *S. aureus* in breast cancer is complex. It may contribute to tumor progression through inflammation, immune modulation, and effects on metastasis, yet certain bacterial products—particularly EVs—can also enhance treatment responses under specific conditions. This dual behavior highlights how strongly its effects depend on context, and why its role in breast cancer biology varies across disease stage, tumor subtype, and microenvironment.

### 2.5. Clostridium Species in Breast Cancer

#### 2.5.1. Overview of *Clostridium Species*

Clinical and microbiome studies increasingly link *Clostridium* species to breast cancer–associated dysbiosis, although their roles appear context dependent. Patients often show shifts in gut microbiota composition, including enrichment of *Clostridiaceae* and reduced overall diversity, suggesting systemic microbial alterations associated with disease [162]. Meta-analyses likewise identify *Clostridium* among the taxa most consistently associated with breast cancer across fecal and tissue samples [162,163]. Functionally, *Clostridium* species may influence tumor risk through metabolic activity. Some strains produce β-glucuronidase, which deconjugates estrogens in the gut and increases circulating levels of bioactive estrogens—an established risk factor for hormone receptor–positive breast cancer [162,164]. Elevated β-glucuronidase activity in patients supports enhanced microbiota-driven estrogen recycling. In addition, *Clostridium*-associated production of secondary bile acids such as deoxycholic acid (DCA) has been linked to oxidative stress, DNA damage, and activation of oncogenic pathways [165]. *Clostridium*-related taxa have also been detected in breast tissue, where differences between malignant and benign samples suggest a role in the tumor microenvironment, although findings are inconsistent across studies [62,102]. Importantly, these bacteria have a dual nature: many commensal strains contribute to gut homeostasis and anti-inflammatory effects, while pathogenic species (e.g., *Clostridioides difficile*, *Clostridium perfringens*) are linked to toxin production and infection, particularly in immunocompromised patients [166]. Rare cases involving *Clostridium septicum* further illustrate how certain species can exploit hypoxic tumor environments [167]. Overall, current evidence suggests that *Clostridium* species contribute to breast cancer primarily through systemic metabolic effects—especially estrogen recycling and bile acid metabolism—rather than direct tumor colonization, highlighting their role within the gut–breast axis.

#### 2.5.2. General Characteristics of *Clostridium Species*

*Clostridium* species are obligate anaerobic, Gram-positive, spore-forming rods that make up a substantial fraction of the gut microbiota (roughly 10–40%). They are widespread in the environment (e.g., soil and water) and commonly colonize the human gastrointestinal tract. The genus is phylogenetically diverse, with clusters IV and XIVa dominating in the gut and contributing to metabolic and immune balance [168,169]. Colonization begins early in life—species such as *C. difficile*, *C. paraputrificum*, and *C. butyricum* can be detected in infancy—and their abundance is influenced by diet and early exposures like breastfeeding [168,169]. Functionally, *Clostridium* species are fermenters that generate short-chain fatty acids (SCFAs), including acetate, propionate, and especially butyrate. Butyrate supports intestinal health by fueling colonocytes, reducing inflammation, lowering luminal pH, and limiting pathogen growth [170]. Consistent with this, beneficial taxa such as *C. butyricum* and *Eubacterium rectale* are associated with improved outcomes in inflammatory bowel disease, while loss of key *Clostridium* groups correlates with gut dysfunction [171]. These bacteria also participate in bile acid metabolism; species such as *C. scindens* and *C. hiranonis* convert primary bile acids into secondary forms like deoxycholic acid (DCA), shaping host metabolism and immune responses [172,173]. In addition, some species (e.g., *C. sporogenes*, *C. cadaveris*) metabolize tryptophan into indolepropionic acid, which supports barrier integrity and has anti-inflammatory effects [174]. Despite these benefits, certain *Clostridium* species are pathogenic. Toxigenic strains such as *Clostridioides difficile* and *Clostridium perfringens* produce potent toxins and can cause severe disease, particularly in immunocompromised individuals, including patients undergoing chemotherapy [169].

#### 2.5.3. Breast–Tumor Association of *Clostridium Species*

Goedert et al. found that postmenopausal breast cancer patients have lower gut microbial diversity than healthy controls, along with shifts in composition—higher levels of taxa such as *Ruminococcaceae*, *Faecalibacterium*, and *Clostridiaceae*, and reduced *Lachnospiraceae* and *Dorea* [162]. Environmental exposures, including tobacco, may further disturb this balance by introducing or selecting for taxa such as *Clostridium* [50]. A large meta-analysis of 48 studies (over 3700 patients) likewise identified *Clostridium* as one of the taxa most consistently associated with breast cancer across both fecal and tissue samples, supporting its link to disease-associated dysbiosis [175]. Among these species, *Clostridium septicum* stands out for its association with malignancy. It can thrive in hypoxic, necrotic tumor environments and produces α-toxin, which damages cell membranes and promotes tissue necrosis. Clinically, it has been linked to serious infections in breast cancer patients, including bacteremia and neutropenic colitis. These features have also led to interest in bacterial toxins as potential tools for targeted cancer therapy [176,177]. Spread from the gut to breast tissue may occur through several routes, including immune cell–mediated transport (the entero-mammary pathway) or translocation into the bloodstream following chemotherapy-induced mucosal injury [8]. Nevertheless, current evidence suggests that *Clostridium* influences breast cancer mainly through systemic effects of its metabolites, rather than direct colonization of tumor tissue.

#### 2.5.4. Pro-Tumor Functions of *Clostridium Species*

A key link between *Clostridium* and breast cancer lies in estrogen metabolism. Some species produce β-glucuronidase, which deconjugates estrogens in the gut and allows them to be reabsorbed into circulation. Higher β-glucuronidase activity has been reported in breast cancer patients, consistent with increased estrogen availability and a role in hormone-driven tumor growth [50,51]. Diet appears to influence this process, as higher fiber intake is associated with lower *Clostridium* abundance and reduced enzyme activity [178]. Both earlier and recent studies point in the same direction. Increased levels of metabolically active *Clostridium paraputrificum* have been linked to changes in bile acid metabolism and higher production of estrogenic compounds such as estradiol and estrone, particularly with high-fat diets [179]. This underscores how diet, microbial metabolism, and cancer risk are closely connected. Beyond systemic effects, some *Clostridium* species may also act locally. For example, *Clostridium histolyticum* produces collagenases that can degrade extracellular matrix, potentially altering tissue structure and tumor–stroma interactions [180]. There is also evidence that *Clostridium* can influence adipocyte function and adipokine signaling, with downstream effects on inflammation and cell growth in breast tissue [175]. These local effects, however, appear to vary depending on tumor subtype and context. Nevertheless, most evidence points to systemic metabolites as the main drivers. One of the best studied is deoxycholic acid (DCA), a secondary bile acid produced by *Clostridium*. DCA can circulate and accumulate in tissues, including the breast, and higher levels—often linked to high-fat diets—have been associated with tumor progression [157,165,181]. Mechanistically, DCA promotes proliferation, induces oxidative stress and DNA damage, and activates pro-tumorigenic pathways such as Wnt/β-catenin signaling, while also shaping inflammatory responses [157,182,183]. Overall, *Clostridium* likely contributes to breast cancer through a mix of metabolic and microenvironmental effects. While many species are beneficial under normal conditions, dysbiosis—particularly shifts that increase estrogen-modulating activity and metabolites like DCA—may favor tumor development. Clarifying strain-specific roles will be important for assessing whether these pathways can be targeted therapeutically.

## 3. Limitations of Breast Cancer Microbiome Studies

Microbiome research has advanced rapidly; however, several important technical and interpretive limitations warrant careful consideration. Contamination is a major concern, particularly in low-biomass samples such as breast tissue, where microbial DNA is often near detection limits [184]. Contaminants introduced during sample collection, DNA extraction, and sequencing reagents (the “kitome”) can produce misleading signals that are difficult to distinguish from true tissue-associated microbes [185,186]. This issue is compounded by inconsistent use of negative controls and variable decontamination strategies across studies. In addition, differences in sampling approaches (e.g., tumor tissue, adjacent normal tissue, nipple aspirate fluid, and skin microbiota) may confound interpretation by capturing external or dermal contamination rather than intrinsic microbial communities. Methodological heterogeneity—including variation in DNA extraction protocols, sequencing platforms, and targeted 16S rRNA regions—further introduces bias and limits reproducibility [187,188]. Reliance on 16S rRNA gene sequencing also restricts taxonomic resolution and provides limited functional insight. Moreover, host and clinical factors such as antibiotic exposure, chemotherapy, hormonal status, diet, and body mass index are not consistently controlled but can significantly influence microbial composition. In particular, breast cancer patients are commonly exposed to broad-spectrum antibiotics during treatment, yet this is often not accounted for in microbiome studies. Antibiotics are known to reduce microbial diversity, deplete beneficial commensals, and promote dysbiosis with long-lasting effects [189,190]. Antibiotic use has also been linked to altered treatment responses and impaired immunotherapy efficacy of cancer patients [191,192]. Failure to control for this major clinical variable can therefore confound associations between microbiome composition and breast cancer. Finally, it remains unresolved whether detected microbial DNA reflects viable, active communities or transient bacterial fragments. Collectively, these limitations highlight the need for standardized protocols, stringent contamination control, and integrated multi-omics and culture-based approaches to more accurately define the microbiome’s role in breast cancer biology.

## 4. Discussion

**Figure 3:**
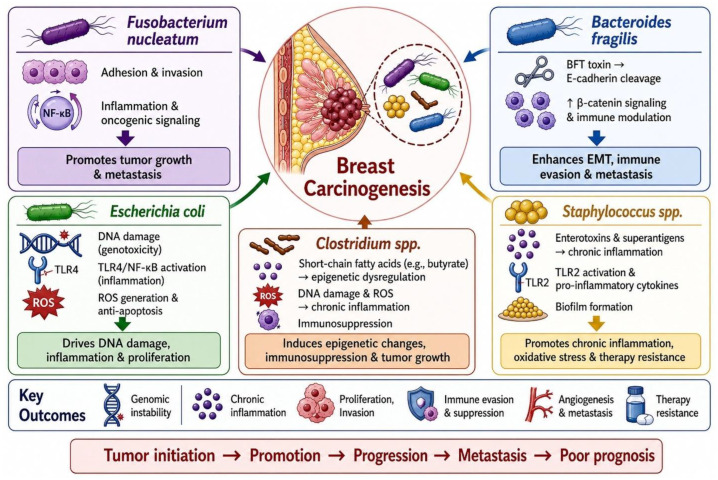
Comparison of pro-tumor roles of five bacteria (*Fusobacterium nucleatum*, *Escherichia coli*, *Bacteroides fragilis*, *Staphylococcus aureus*, and *Clostridium* sp.) in breast cancer. The figure was created by ChatGPT (https://chatgpt.com/).

This review highlights the growing evidence that the breast microbiota contributes to both cancer initiation and progression, focusing on five key bacterial groups (Figure 3). A common theme is their ability to create a pro-inflammatory tumor environment. For instance, *Fusobacterium nucleatum* activates NF-κB signaling and drives IL-1β production [59], while *Bacteroides fragilis* toxin promotes reactive oxygen species (ROS) and cytokines such as IL-6, IL-8, and IL-17 [117,126,127]. *Escherichia coli* also contributes through secreted factors that stimulate cytokine release and stromal activation [43]. In addition to inflammation, some bacteria directly affect genomic stability. A well-established example is *pks*-positive *E. coli*, which produces colibactin and induces DNA double-strand breaks. Although similar direct genotoxic effects are less defined for other taxa, their ability to increase oxidative stress and interfere with DNA repair suggests indirect roles in mutagenesis [25,94].

Immune modulation is another key mechanism. *F. nucleatum* can suppress anti-tumor immunity through virulence factors such as Fap2 and RadD, which inhibit NK and T-cell activity via TIGIT and Siglec-7 interactions (Table 1) [30,38,39,57]. It can also promote PD-L1 expression through NF-κB signaling, reducing CD8^+^ T-cell function. *Staphylococcus aureus* similarly influences immune responses through TLR2 signaling and context-dependent PD-L1 regulation [49]. Together, these effects reshape the immune landscape in ways that favor tumor survival and may limit responses to immunotherapy. Metabolic reprogramming also plays a central role. Microbial products—including short-chain fatty acids (SCFAs), bile acid derivatives, and enzymes involved in estrogen metabolism—can significantly influence tumor biology. *Clostridium* species, for example, increase estrogen reactivation via β-glucuronidase, while secondary bile acids such as deoxycholic acid (DCA) activate pathways like Wnt/β-catenin [51]. Metabolites from *E. coli* and *F. nucleatum* further support tumor growth and can contribute to therapy resistance [3,21,77]. These effects highlight how microbial metabolism extends beyond the gut to shape systemic cancer risk and progression.

Importantly, these microbial effects are not uniform. Their impact depends on the specific strain and the host context. *S. aureus*, for example, has been linked to both tumor-promoting processes, such as metastasis through neutrophil extracellular traps, and tumor-suppressive effects, including increased sensitivity to tamoxifen. Likewise, *Clostridium* species can support gut homeostasis under normal conditions but contribute to tumorigenesis when dysregulated. This growing body of work points to several clinical opportunities. Microbial signatures—such as enrichment of *F. nucleatum*, *pks*-positive *E. coli*, and *Clostridium*—may serve as non-invasive biomarkers for early detection or risk assessment. The microbiome may also help explain differences in treatment response; for example, microbial effects on PD-L1 expression or DNA repair pathways could influence sensitivity to immunotherapy or chemotherapy. In addition, certain microbial products may enhance treatment efficacy. These insights raise the possibility of using microbiome profiling to guide more personalized cancer therapy. Targeting the microbiome is another area of interest. Strategies such as selective antibiotics, probiotics, dietary modification, or engineered bacteria could potentially retard tumor progression or promote anti-tumor immunity. Enzymes like β-glucuronidase are also being considered as therapeutic targets to limit estrogen-driven tumor growth. Nevertheless, moving these ideas into the clinic would require more advancements in research. Larger, well-controlled human studies would be needed to establish causality of the breast cancer microbiota, along with standardized methods and a clearer understanding of which microbial functions are the most relevant to breast cancer progression versus treatment.

## 5. Conclusions

In summary, the breast cancer microbiome is an emerging and complex contributor to tumor biology, with evidence linking it to carcinogenesis, progression, immune modulation, and treatment response. For example, *Fusobacterium nucleatum* promotes immune evasion by inhibiting NK and T cell activity and fostering an immunosuppressive tumor microenvironment, supporting disease progression [25]. Certain strains of *Escherichia coli* produce colibactin, a genotoxin that induces DNA damage and genomic instability, contributing to carcinogenesis [25]. *Bacteroides fragilis* (particularly enterotoxigenic strains) drives inflammation via toxin-mediated activation of NF-κB and STAT3 signaling, while *Clostridium* species generate metabolites and inflammatory signals that can promote tumor-supportive conditions [1]. Members of the *Staphylococcus* genus, commonly present in breast tissue, can modulate local immune responses through toxin production and biofilm formation, with context-dependent effects on tumor biology [26]. Despite the growing body of evidence on the breast cancer microbiome, the field remains limited by incomplete mechanistic resolution and a lack of integrated functional and structural data. Key questions remain regarding causality, spatial organization, and functional activity within breast tissue. A more mechanistic understanding of host–microbe interactions is needed to separate true biological effects from confounding noise. Integrating multi-omics, spatial profiling, and functional validation will enhance precision oncology based on breast cancer microbiome, allowing for more personalized approaches to breast cancer management.

## Figures and Tables

**Table 1:** Bacterial Components and Virulence Factors Contributing to Breast Cancer Initiation and Progression.

Bacteria	Bacterial Component	Mechanisms/Virulence Factors	Role in Breast Cancer Initiation/Progression	Evidence (Studies/Models)	Ref.
*F. nucleatum*	Fap2	An autotransporter protein 2 which targets Gal–GalNAc moieties that are displayed on breast cancer cells	Increase attachment to malignant breast cancer and fusobacterial-driven metastasis by suppressing antitumor immunity of T lymphocytes and NK cells	Mouse models	[21]
	FadA	An outer membrane protein which binds to E-cadherin on breast epithelial cells	*Fn* accelerates the development of breast cancer by triggering the Wnt/β-catenin signaling pathway	Human cell lines	[38]
	RadD	An autotransporter protein functions as a bacterial ligand for Siglec-7.	Impair NK cell–dependent tumor immune surveillance.	Human cell lines	[39]
	Extracellular vesicles (EVs)	*Fn-derived* gDNA that regulate TLR4	Enhanced the viability, proliferation, migration, and invasion of breast cancer cells by regulating TLR4	Breast tissues of breast cancer patients	[27]
	Lipopolysaccharide	TLR4 stimulation	Promotes NF-κβ activation and the expression of inflammatory and anti-apoptotic proteins such as Bcl-2 and Bcl-Xl.	Microbiome study in breast tissue	[39]
	Microbial metabolites (succinic acid, formate, ADP-heptose and butyrate acid)		Fuel tumor progression, immune evasion, metastasis and therapy resistance	Microbiome study in breast tissue	[30]
*E. coli*	Colibactin	A genotoxin that can form inter-strand crosslinks in DNA	Inducing double-strand breaks that could cause carcinogenesis and growth advantage to breast cancer	Microbiome studies in human cell lines	[40,41,42]
	Secretome of *E. coli*	Specific bacterial metabolites secreted by *E. coli*	Induces breast stromal fibroblasts to produce pro-carcinogenic/pro-inflammatory cytokines	In vitro, cell line models	[43]
	N-acetyl-L-methionine, nicotinamide riboside, N-acetylneuraminic acid, mannose-1-phosphate, and glutathionylspermidine.	*E. coli* metabolites associated with breast cancer progression	Modulate energy metabolism and induce chemotherapy resistance of breast cancer cells to promote their growth and survival.	In vitro, cell line models	[44]
*B. fragilis*	*B. fragilis* toxin (BFT)	A heat-labile enterotoxins linked with inducing inflammation	Enlarge and thicken breast duct lining and increase stromal infiltration, collagen deposition, hyperplasia, and T-cell infiltration	Mouse model	[45]
			ROS-mediated oxidative stress link microbial-driven inflammation to breast cancer progression.	In vitro, cell lines	[46]
			Involves the activation of multiple pro-inflammatory cytokines including IL-8, plays a key role in creating a microenvironment that increases breast cancer	In vitro, cell lines	[47]
*Staphylococcus* spp.	Injected bacteria	Neutrophil extracellular traps (NETs)	Recruited neutrophils form NETs which trapped circulating tumor cells and promoted metastasis in the breast tissue	Mouse models	[48]
	Extracellular vesicles (EV’s)	Function as estrobolomes	Decrease p-ERK and p-AKT to increased death of the breast cancer cells	Mouse model	[48]
	Co culture of *S. aureus* with cells plus IFN-γ	Increased TLR2 expression	Possibly amplifying the tumorigenicity of the cells	TNBC cell lines	[49]
*Clostridium* spp.	β-glucuronidase	Deconjugates glucuronidated estrogens, allowing the reabsorption of reactivated estrogens	Increased oestrogen bioavailability promotes estrogen-driven Tumor growth	Breast cancer survivors	[50,51]
	7α-dehydroxylation pathway	Facilitates the transformation of primary bile acids into deoxycholic acid (DCA)	Activate oncogenic pathways such as Wnt/β-catenin signaling thereby creating a pro-tumorigenic microenvironment in breast tissues	Cell lines	[51]

## Data Availability

No new data were created or analyzed in this study. Data sharing is not applicable to this article.

## References

[1] German R., Marino N., Hemmerich C., Podicheti R., Rusch D.B., Stiemsma L.T., Gao H., Xuei X., Rockey P., Storniolo A.M. (2023). Exploring breast tissue microbial composition and the association with breast cancer risk factors. Breast Cancer Res..

[2] Van der Merwe M., Van Niekerk G., Botha A., Engelbrecht A.M. (2021). The onco-immunological implications of *Fusobacterium nucleatum* in breast cancer. Immunol. Lett..

[3] Guo J., Zhu P., Li J., Xu L., Tang Y., Liu X., Guo S., Xia J. (2024). *Fusobacterium nucleatum* promotes PD-L1 expression in cancer cells to evade CD8^+^ T cell killing in breast cancer. Hum. Immunol..

[4] Geng S., Xiang T., Shi Y., Cao M., Wang D., Wang J., Li X., Song H., Zhang Z., Shi J. (2025). Locally producing antibacterial peptide to deplete intratumoral pathogen for preventing metastatic breast cancer. Acta Pharm. Sin. B.

[5] Fu A., Yao B., Dong T., Chen Y., Yao J., Liu Y., Li H., Bai H., Liu X., Zhang Y. (2022). Tumor-resident intracellular microbiota promotes metastatic colonization in breast cancer. Cell.

[6] Wang J., Xu D., Hu S., Zheng B., Chen Y., Pan T. (2025). The Impact of Microbiome on Breast Cancer and Regulatory Strategies. Microorganisms.

[7] Zhang J., Xia Y., Sun J. (2021). Breast and gut microbiome in health and cancer. Genes Dis..

[8] Selvamani S., Dailin D.J., Gupta V.K., Wahid M., Keat H.C., Natasya K.H., Malek R.A., Haque S., Sayyed R.Z., Abomoelak B. (2021). An Insight into Probiotics Bio-Route: Translocation from the Mother’s Gut to the Mammary Gland. Appl. Sci..

[9] Rodríguez J.M., Fernández L., Verhasselt V. (2021). The Gut-Breast Axis: Programming Health for Life. Nutrients.

[10] Flood T.R., Kuennen M.R., Blacker S.D., Myers S.D., Walker E.F., Lee B.J. (2022). The effect of sex, menstrual cycle phase and oral contraceptive use on intestinal permeability and ex-vivo monocyte TNFα release following treatment with lipopolysaccharide and hyperthermia. Cytokine.

[11] Atashgaran V., Wrin J., Barry S.C., Dasari P., Ingman W.V. (2016). Dissecting the Biology of Menstrual Cycle-Associated Breast Cancer Risk. Front. Oncol..

[12] Schaadt N.S., Alfonso J.C.L., Schönmeyer R., Grote A., Forestier G., Wemmert C., Krönke N., Stoeckelhuber M., Kreipe H.H., Hatzikirou H. (2017). Image analysis of immune cell patterns in the human mammary gland during the menstrual cycle refines lymphocytic lobulitis. Breast Cancer Res. Treat..

[13] Farache J., Koren I., Milo I., Gurevich I., Kim K.W., Zigmond E., Furtado G.C., Lira S.A., Shakhar G. (2013). Luminal bacteria recruit CD103+ dendritic cells into the intestinal epithelium to sample bacterial antigens for presentation. Immunity.

[14] Soto-Pantoja D.R., Gaber M., Arnone A.A., Bronson S.M., Cruz-Diaz N., Wilson A.S., Clear K.Y.J., Ramirez M.U., Kucera G.L., Levine E.A. (2021). Diet Alters Entero-Mammary Signaling to Regulate the Breast Microbiome and Tumorigenesis. Cancer Res..

[15] Favier C.F., Vaughan E.E., Vos W.M.D., Akkermans A.D.L. (2002). Molecular Monitoring of Succession of Bacterial Communities in Human Neonates. Appl. Environ. Microbiol..

[16] Fernández L., Langa S., Martín V., Maldonado A., Jiménez E., Martín R., Rodríguez J.M. (2013). The human milk microbiota: Origin and potential roles in health and disease. Pharmacol. Res..

[17] Bernardo G., Le Noci V., Di Modica M., Montanari E., Triulzi T., Pupa S.M., Tagliabue E., Sommariva M., Sfondrini L. (2023). The Emerging Role of the Microbiota in Breast Cancer Progression. Cells.

[18] Furuta S. (2024). Microbiome-Stealth Regulator of Breast Homeostasis and Cancer Metastasis. Cancers.

[19] Rescigno M., Urbano M., Valzasina B., Francolini M., Rotta G., Bonasio R., Granucci F., Kraehenbuhl J.-P., Ricciardi-Castagnoli P. (2001). Dendritic cells express tight junction proteins and penetrate gut epithelial monolayers to sample bacteria. Nat. Immunol..

[20] Vazquez-Torres A., Jones-Carson J., Bäumler A.J., Falkow S., Valdivia R., Brown W., Le M., Berggren R., Parks W.T., Fang F.C. (1999). Extraintestinal dissemination of Salmonella by CD18-expressing phagocytes. Nature.

[21] Parhi L., Alon-Maimon T., Sol A., Nejman D., Shhadeh A., Fainsod-Levi T., Yajuk O., Isaacson B., Abed J., Maalouf N. (2020). Breast cancer colonization by *Fusobacterium nucleatum* accelerates tumor growth and metastatic progression. Nat. Commun..

[22] Zhou S., Yu J. (2023). Crohn’s disease and breast cancer: A literature review of the mechanisms and treatment. Intern. Emerg. Med..

[23] Wexler H.M. (2007). Bacteroides: The good, the bad, and the nitty-gritty. Clin. Microbiol. Rev..

[24] Ukraincev N.I., Kashutina M.I., Kasatkina L.I., Abduraimov A.B., Zhernov Y.V. (2025). Mammary Gland Microbiota in Benign Breast Diseases. Int. J. Mol. Sci..

[25] Miyasaka T., Yamada T., Uehara K., Sonoda H., Matsuda A., Shinji S., Ohta R., Kuriyama S., Yokoyama Y., Takahashi G. (2024). Pks-positive *Escherichia coli* in tumor tissue and surrounding normal mucosal tissue of colorectal cancer patients. Cancer Sci..

[26] Touaitia R., Mairi A., Ibrahim N.A., Basher N.S., Idres T., Touati A. (2025). Staphylococcus aureus: A Review of the Pathogenesis and Virulence Mechanisms. Antibiotics.

[27] Li G., Sun Y., Huang Y., Lian J., Wu S., Luo D., Gong H. (2023). *Fusobacterium nucleatum*-derived small extracellular vesicles facilitate tumor growth and metastasis via TLR4 in breast cancer. BMC Cancer.

[28] Zhao F., An R., Ma Y., Yu S., Gao Y., Wang Y., Yu H., Xie X., Zhang J. (2025). Integrated spatial multi-omics profiling of *Fusobacterium nucleatum* in breast cancer unveils its role in tumour microenvironment modulation and cancer progression. Clin. Transl. Med..

[29] Geng S., Guo P., Li X., Shi Y., Wang J., Cao M., Zhang Y., Zhang K., Li A., Song H. (2024). Biomimetic Nanovehicle-Enabled Targeted Depletion of Intratumoral *Fusobacterium nucleatum* Synergizes with PD-L1 Blockade against Breast Cancer. ACS Nano.

[30] Jiang S.S., Chen Y.X., Fang J.Y. (2025). *Fusobacterium nucleatum*: Ecology, pathogenesis and clinical implications. Nat. Rev. Microbiol..

[31] Tierney B.T., Yang Z., Luber J.M., Beaudin M., Wibowo M.C., Baek C., Mehlenbacher E., Patel C.J., Kostic A.D. (2019). The Landscape of Genetic Content in the Gut and Oral Human Microbiome. Cell Host Microbe.

[32] Rubinstein M.R., Wang X., Liu W., Hao Y., Cai G., Han Y.W. (2013). *Fusobacterium nucleatum* promotes colorectal carcinogenesis by modulating E-cadherin/β-catenin signaling via its FadA adhesin. Cell Host Microbe.

[33] Abed J., Emgård J.E., Zamir G., Faroja M., Almogy G., Grenov A., Sol A., Naor R., Pikarsky E., Atlan K.A. (2016). Fap2 Mediates *Fusobacterium nucleatum* Colorectal Adenocarcinoma Enrichment by Binding to Tumor-Expressed Gal-GalNAc. Cell Host Microbe.

[34] Schöpf F., Marongiu G.L., Milaj K., Sprink T., Kikhney J., Moter A., Roderer D. (2025). Structural basis of *Fusobacterium nucleatum* adhesin Fap2 interaction with receptors on cancer and immune cells. Nat. Commun..

[35] Bullman S., Pedamallu C.S., Sicinska E., Clancy T.E., Zhang X., Cai D., Neuberg D., Huang K., Guevara F., Nelson T. (2017). Analysis of Fusobacterium persistence and antibiotic response in colorectal cancer. Science.

[36] Kostic A.D., Chun E., Robertson L., Glickman J.N., Gallini C.A., Michaud M., Clancy T.E., Chung D.C., Lochhead P., Hold G.L. (2013). *Fusobacterium nucleatum* potentiates intestinal tumorigenesis and modulates the tumor-immune microenvironment. Cell Host Microbe.

[37] Yang Y., Jobin C. (2020). A mutational signature that can be made by a bacterium arises in human colon cancer. Nature.

[38] Wang X., He X., Zhong B. (2024). Oral microbiota: The overlooked catalyst in cancer initiation and progression. Front. Cell Dev. Biol..

[39] Galaski J., Rishiq A., Liu M., Bsoul R., Bergson A., Lux R., Bachrach G., Mandelboim O. (2024). *Fusobacterium nucleatum* subsp. nucleatum RadD binds Siglec-7 and inhibits NK cell-mediated cancer cell killing. iScience.

[40] Feng T., Xie F., Lee L.M.Y., Lin Z., Tu Y., Lyu Y., Yu P., Wu J., Chen B., Zhang G. (2025). Cellular senescence in cancer: From mechanism paradoxes to precision therapeutics. Mol. Cancer.

[41] Heaton L., Figard S.D., Garcia R.A. (2024). B-351 Genotoxic Producing *Escherichia coli* Protects and Promotes Breast Adenocarcinoma. Clin. Chem..

[42] Secher T., Samba-Louaka A., Oswald E., Nougayrède J.P. (2013). *Escherichia coli* producing colibactin triggers premature and transmissible senescence in mammalian cells. PLoS ONE.

[43] Alshehri J.H., Al-Nasrallah H.K., Al-Ansari M.M., Aboussekhra A. (2024). Activation of Mammary Epithelial and Stromal Fibroblasts upon Exposure to *Escherichia coli* Metabolites. Cells.

[44] AlMalki R.H., Sebaa R., Al-Ansari M.M., Al-Alwan M., Alwehaibi M.A., Rahman A.M. (2023). A. *E. coli* Secretome Metabolically Modulates MDA-MB-231 Breast Cancer Cells’ Energy Metabolism. Int. J. Mol. Sci..

[45] Parida S., Wu S., Siddharth S., Wang G., Muniraj N., Nagalingam A., Hum C., Mistriotis P., Hao H., Talbot C.C. (2021). A Procarcinogenic Colon Microbe Promotes Breast Tumorigenesis and Metastatic Progression and Concomitantly Activates Notch and β-Catenin Axes. Cancer Discov..

[46] Malla R., Surepalli N., Farran B., Malhotra S.V., Nagaraju G.P. (2021). Reactive oxygen species (ROS): Critical roles in breast tumor microenvironment. Crit. Rev. Oncol. Hematol..

[47] Baggiolini M., Clark-Lewis I. (1992). Interleukin-8, a chemotactic and inflammatory cytokine. FEBS Lett..

[48] Qi J.L., He J.R., Liu C.B., Jin S.M., Gao R.Y., Yang X., Bai H.M., Ma Y.B. (2020). Pulmonary Staphylococcus aureus infection regulates breast cancer cell metastasis via neutrophil extracellular traps (NETs) formation. MedComm.

[49] Rad S.K., Li R., Yeo K.K.L., Cooksley C., Shaghayegh G., Vreugde S., Wu F., Tomita Y., Price T.J., Ingman W.V. (2025). Cell Line-Dependent Internalization, Persistence, and Immunomodulatory Effects of Staphylococcus aureus in Triple-Negative Breast Cancer. Cancers.

[50] Liu Y., Ning H., Li Y., Li Y., Ma J. (2025). The microbiota in breast cancer: Dysbiosis, microbial metabolites, and therapeutic implications. Am. J. Cancer Res..

[51] Sui Y., Wu J., Chen J. (2021). The Role of Gut Microbial beta-Glucuronidase in Estrogen Reactivation and Breast Cancer. Front. Cell Dev. Biol..

[52] Little A., Tangney M., Tunney M.M., Buckley N.E. (2023). *Fusobacterium nucleatum*: A novel immune modulator in breast cancer?. Expert Rev. Mol. Med..

[53] Pavitra E., Kancharla J., Gupta V.K., Prasad K., Sung J.Y., Kim J., Tej M.B., Choi R., Lee J.H., Han Y.K. (2023). The role of NF-kappaB in breast cancer initiation, growth, metastasis, and resistance to chemotherapy. BioMed Pharmacother..

[54] Gaba F.I., González R.C., Martïnez R.G. (2022). The Role of Oral *Fusobacterium nucleatum* in Female Breast Cancer: A Systematic Review and Meta-Analysis. Int. J. Dent..

[55] Guo X., Yu K., Huang R. (2024). The ways *Fusobacterium nucleatum* translocate to breast tissue and contribute to breast cancer development. Mol. Oral Microbiol..

[56] Despins C.A., Brown S.D., Robinson A.V., Mungall A.J., Allen-Vercoe E., Holt R.A. (2021). Modulation of the Host Cell Transcriptome and Epigenome by *Fusobacterium nucleatum*. mBio.

[57] Huang Y., Guo Z., Zeng Z., Shang C., Zhang Y., Ran Z., Luo G., Shen S., Liu Y., Zhou P. (2025). *Fusobacterium nucleatum* promotes metastasis of breast cancer via the miR-21-3p/FOXO3 axis. Front. Oncol..

[58] Pires B.R., Mencalha A.L., Ferreira G.M., de Souza W.F., Morgado-Diaz J.A., Maia A.M., Correa S., Abdelhay E.S. (2017). NF-kappaB Is Involved in the Regulation of EMT Genes in Breast Cancer Cells. PLoS ONE.

[59] Chang C.M., Lam L.Y., Lam H.Y.P., Kao P.Y., Hsu S.T., Wu W.J., Chang K.C., Huang C.Y. (2025). Potential role of intratumoral *Fusobacterium nucleatum* and interleukin-1 beta in breast cancer cell growth. J. Microbiol. Immunol. Infect..

[60] Gur C., Ibrahim Y., Isaacson B., Yamin R., Abed J., Gamliel M., Enk J., Bar-On Y., Stanietsky-Kaynan N., Coppenhagen-Glazer S. (2015). Binding of the Fap2 protein of *Fusobacterium nucleatum* to human inhibitory receptor TIGIT protects tumors from immune cell attack. Immunity.

[61] Parida S., Nandi D., Verma D., Yi M., Yende A., Queen J., Gabrielson K.L., Sears C.L., Sharma D. (2026). A pro-carcinogenic oral microbe internalized by breast cancer cells promotes mammary tumorigenesis. Cell Commun. Signal..

[62] Urbaniak C., Gloor G.B., Brackstone M., Scott L., Tangney M., Reid G. (2016). The Microbiota of Breast Tissue and Its Association with Breast Cancer. Appl. Environ. Microbiol..

[63] Manzoor H., Jabeen I., Saeed M.T., Kayani M.U.R., Huang L. (2025). Metagenomic analyses reveal *E. coli*-derived siderophores as potential signatures for breast cancer. J. Transl. Med..

[64] Goodwin A.C., Destefano Shields C.E., Wu S., Huso D.L., Wu X., Murray-Stewart T.R., Hacker-Prietz A., Rabizadeh S., Woster P.M., Sears C.L. (2011). Polyamine catabolism contributes to enterotoxigenic Bacteroides fragilis-induced colon tumorigenesis. Proc. Natl. Acad. Sci. USA.

[65] Tenaillon O., Skurnik D., Picard B., Denamur E. (2010). The population genetics of commensal *Escherichia coli*. Nat. Rev. Microbiol..

[66] Eckburg P.B., Bik E.M., Bernstein C.N., Purdom E., Dethlefsen L., Sargent M., Gill S.R., Nelson K.E., Relman D.A. (2005). Diversity of the human intestinal microbial flora. Science.

[67] Martinson J.N.V., Walk S.T. (2020). *Escherichia coli* Residency in the Gut of Healthy Human Adults. EcoSal Plus.

[68] Bentley R., Meganathan R. (1982). Biosynthesis of vitamin K (menaquinone) in bacteria. Microbiol. Rev..

[69] Hudault S., Guignot J., Servin A.L. (2001). *Escherichia coli* strains colonising the gastrointestinal tract protect germfree mice against *Salmonella typhimurium* infection. Gut.

[70] Reid G., Howard J., Gan B.S. (2001). Can bacterial interference prevent infection?. Trends Microbiol..

[71] Budiardjo S.J., Stevens J.J., Calkins A.L., Ikujuni A.P., Wimalasena V.K., Firlar E., Case D.A., Biteen J.S., Kaelber J.T., Slusky J.S.G. (2022). Colicin E1 opens its hinge to plug TolC. eLife.

[72] Russell J.B., Jarvis G.N. (2001). Practical mechanisms for interrupting the oral-fecal lifecycle of *Escherichia coli*. J. Mol. Microbiol. Biotechnol..

[73] Montealegre M.C., Roy S., Böni F., Hossain M.I., Navab-Daneshmand T., Caduff L., Faruque A.S.G., Islam M.A., Julian T.R. (2018). Risk Factors for Detection, Survival, and Growth of Antibiotic-Resistant and Pathogenic *Escherichia coli* in Household Soils in Rural Bangladesh. Appl. Environ. Microbiol..

[74] Alhadlaq M.A., Aljurayyad O.I., Almansour A., Al-Akeel S.I., Alzahrani K.O., Alsalman S.A., Yahya R., Al-Hindi R.R., Hakami M.A., Alshahrani S.D. (2024). Overview of pathogenic *Escherichia coli*, with a focus on Shiga toxin-producing serotypes, global outbreaks (1982–2024) and food safety criteria. Gut Pathog..

[75] Abri R., Javadi A., Asghari R., Razavilar V., Salehi T.Z., Safaeeyan F., Rezaee M.A. (2019). Surveillance for enterotoxigenic & enteropathogenic *Escherichia coli* isolates from animal source foods in Northwest Iran. Indian J. Med. Res..

[76] Carlos C., Pires M.M., Stoppe N.C., Hachich E.M., Sato M.I.Z., Gomes T.A.T., Amaral L.A., Ottoboni L.M.M. (2010). *Escherichia coli* phylogenetic group determination and its application in the identification of the major animal source of fecal contamination. BMC Microbiol..

[77] Filippou C., Themistocleous S.C., Marangos G., Panayiotou Y., Fyrilla M., Kousparou C.A., Pana Z.D., Tsioutis C., Johnson E.O., Yiallouris A. (2024). Microbial Therapy and Breast Cancer Management: Exploring Mechanisms, Clinical Efficacy, and Integration within the One Health Approach. Int. J. Mol. Sci..

[78] Johnson J.R., Johnston B., Kuskowski M.A., Nougayrede J.P., Oswald E. (2008). Molecular epidemiology and phylogenetic distribution of the *Escherichia coli* pks genomic island. J. Clin. Microbiol..

[79] Bakthavatchalu V., Wert K.J., Feng Y., Mannion A., Ge Z., Garcia A., Scott K.E., Caron T.J., Madden C.M., Jacobsen J.T. (2018). Cytotoxic *Escherichia coli* strains encoding colibactin isolated from immunocompromised mice with urosepsis and meningitis. PLoS ONE.

[80] Wassenaar T.M. (2018). *E. coli* and colorectal cancer: A complex relationship that deserves a critical mindset. Crit. Rev. Microbiol..

[81] Javed S., Mirani Z.A., Pirzada Z.A. (2021). Phylogenetic Group B2 Expressed Significant Biofilm Formation among Drug Resistant Uropathogenic *Escherichia coli*. Libyan J. Med..

[82] Nicolas-Chanoine M.H., Bertrand X., Madec J.Y. (2014). *Escherichia coli* ST131, an intriguing clonal group. Clin. Microbiol. Rev..

[83] Johnson J.R., Russo T.A. (2002). Extraintestinal pathogenic *Escherichia coli*: “the other bad *E coli*”. J. Lab. Clin. Med..

[84] Pitout J.D. (2012). Extraintestinal pathogenic *Escherichia coli*: An update on antimicrobial resistance, laboratory diagnosis and treatment. Expert Rev. Anti Infect. Ther..

[85] Nowrouzian F.L., Ostblom A.E., Wold A.E., Adlerberth I. (2009). Phylogenetic group B2 *Escherichia coli* strains from the bowel microbiota of Pakistani infants carry few virulence genes and lack the capacity for long-term persistence. Clin. Microbiol. Infect..

[86] Nougayrède J.P., Homburg S., Taieb F., Boury M., Brzuszkiewicz E., Gottschalk G., Buchrieser C., Hacker J., Dobrindt U., Oswald E. (2006). *Escherichia coli* induces DNA double-strand breaks in eukaryotic cells. Science.

[87] Rozani S., Lykoudis P.M. (2024). The impact of intestinal and mammary microbiomes on breast cancer development: A review on the microbiota and oestrobolome roles in tumour microenvironments. Am. J. Surg..

[88] Gustafsson J.K., Davis J.E., Rappai T., McDonald K.G., Kulkarni D.H., Knoop K.A., Hogan S.P., Fitzpatrick J.A., Lencer W.I., Newberry R.D. (2021). Intestinal goblet cells sample and deliver lumenal antigens by regulated endocytic uptake and transcytosis. eLife.

[89] Knoop K.A., Coughlin P.E., Floyd A.N., Ndao I.M., Hall-Moore C., Shaikh N., Gasparrini A.J., Rusconi B., Escobedo M., Good M. (2020). Maternal activation of the EGFR prevents translocation of gut-residing pathogenic *Escherichia coli* in a model of late-onset neonatal sepsis. Proc. Natl. Acad. Sci. USA.

[90] Alexander J.W., Boyce S.T., Babcock G.F., Gianotti L., Peck M.D., Dunn D.L., Pyles T., Childress C.P., Ash S.K. (1990). The process of microbial translocation. Ann. Surg..

[91] Bachmann N.L., Katouli M., Polkinghorne A. (2015). Genomic Comparison of Translocating and Non-Translocating *Escherichia coli*. PLoS ONE.

[92] Asgari B., Burke J.R., Quigley B.L., Bradford G., Hatje E., Kuballa A., Katouli M. (2024). Identification of Virulence Genes Associated with Pathogenicity of Translocating *Escherichia coli* with Special Reference to the Type 6 Secretion System. Microorganisms.

[93] Bradley A.J., Green M.J. (2001). Adaptation of *Escherichia coli* to the bovine mammary gland. J. Clin. Microbiol..

[94] Wernke K.M., Xue M., Tirla A., Kim C.S., Crawford J.M., Herzon S.B. (2020). Structure and bioactivity of colibactin. Bioorganic Med. Chem. Lett..

[95] de Oliveira Alves N., Dalmasso G., Nikitina D., Vaysse A., Ruez R., Ledoux L., Pedron T., Bergsten E., Boulard O., Autier L. (2024). The colibactin-producing *Escherichia coli* alters the tumor microenvironment to immunosuppressive lipid overload facilitating colorectal cancer progression and chemoresistance. Gut Microbes.

[96] Sogari A., Rovera E., Grasso G., Mariella E., Reilly N.M., Lamba S., Mauri G., Durinikova E., Vitiello P.P., Lorenzato A. (2024). Tolerance to colibactin correlates with homologous recombination proficiency and resistance to irinotecan in colorectal cancer cells. Cell Rep. Med..

[97] Raisch J., Rolhion N., Dubois A., Darfeuille-Michaud A., Bringer M.-A. (2015). Intracellular colon cancer-associated *Escherichia coli* promote protumoral activities of human macrophages by inducing sustained COX-2 expression. Lab. Investig..

[98] Nguyen D.-H., Chong A., Hong Y., Min J.-J. (2023). Bioengineering of bacteria for cancer immunotherapy. Nat. Commun..

[99] Altenhoefer A., Oswald S., Sonnenborn U., Enders C., Schulze J., Hacker J., Oelschlaeger T.A. (2004). The probiotic *Escherichia coli* strain Nissle 1917 interferes with invasion of human intestinal epithelial cells by different enteroinvasive bacterial pathogens. FEMS Immunol. Med. Microbiol..

[100] Gurbatri C.R., Radford G.A., Vrbanac L., Im J., Thomas E.M., Coker C., Taylor S.R., Jang Y., Sivan A., Rhee K. (2024). Engineering tumor-colonizing *E. coli* Nissle 1917 for detection and treatment of colorectal neoplasia. Nat. Commun..

[101] Rosenthal E.L., Chung T.K., Parker W.B., Allan P.W., Clemons L., Lowman D., Hong J., Hunt F.R., Richman J., Conry R.M. (2015). Phase I dose-escalating trial of *Escherichia coli* purine nucleoside phosphorylase and fludarabine gene therapy for advanced solid tumors. Ann. Oncol..

[102] Hieken T.J., Chen J., Hoskin T.L., Walther-Antonio M., Johnson S., Ramaker S., Xiao J., Radisky D.C., Knutson K.L., Kalari K.R. (2016). The Microbiome of Aseptically Collected Human Breast Tissue in Benign and Malignant Disease. Sci. Rep..

[103] Nejman D., Livyatan I., Fuks G., Gavert N., Zwang Y., Geller L.T., Rotter-Maskowitz A., Weiser R., Mallel G., Gigi E. (2020). The human tumor microbiome is composed of tumor type-specific intracellular bacteria. Science.

[104] Wu S., Rhee K.-J., Albesiano E., Rabizadeh S., Wu X., Yen H.-R., Huso D.L., Brancati F.L., Wick E., McAllister F. (2009). A human colonic commensal promotes colon tumorigenesis via activation of T helper type 17 T cell responses. Nat. Med..

[105] Lee C.G., Hwang S., Gwon S.Y., Park C., Jo M., Hong J.E., Rhee K.J. (2022). Bacteroides fragilis Toxin Induces Intestinal Epithelial Cell Secretion of Interleukin-8 by the E-Cadherin/β-Catenin/NF-κB Dependent Pathway. Biomedicines.

[106] Elsaghir H., Reddivari A.K.R. (2025). Bacteroides Fragilis. StatPearls.

[107] Arumugam M., Raes J., Pelletier E., Le Paslier D., Yamada T., Mende D.R., Fernandes G.R., Tap J., Bruls T., Batto J.M. (2011). Enterotypes of the human gut microbiome. Nature.

[108] Patrick S. (2022). A tale of two habitats: Bacteroides fragilis, a lethal pathogen and resident in the human gastrointestinal microbiome. Microbiology.

[109] Zitomersky N.L., Coyne M.J., Comstock L.E. (2011). Longitudinal analysis of the prevalence, maintenance, and IgA response to species of the order Bacteroidales in the human gut. Infect. Immun..

[110] Sanfilippo L., Li C.K., Seth R., Balwin T.J., Menozzi M.G., Mahida Y.R. (2000). Bacteroides fragilis enterotoxin induces the expression of IL-8 and transforming growth factor-beta (TGF-beta) by human colonic epithelial cells. Clin. Exp. Immunol..

[111] Wu S., Powell J., Mathioudakis N., Kane S., Fernandez E., Sears C.L. (2004). Bacteroides fragilis enterotoxin induces intestinal epithelial cell secretion of interleukin-8 through mitogen-activated protein kinases and a tyrosine kinase-regulated nuclear factor-kappaB pathway. Infect. Immun..

[112] Boleij A., Hechenbleikner E.M., Goodwin A.C., Badani R., Stein E.M., Lazarev M.G., Ellis B., Carroll K.C., Albesiano E., Wick E.C. (2015). The Bacteroides fragilis toxin gene is prevalent in the colon mucosa of colorectal cancer patients. Clin. Infect. Dis..

[113] Sears C.L., Garrett W.S. (2014). Microbes, microbiota, and colon cancer. Cell Host Microbe.

[114] Méndez-López L.F. (2022). Revisiting Epithelial Carcinogenesis. Int. J. Mol. Sci..

[115] Casero R.A., Pegg A.E. (2009). Polyamine catabolism and disease. Biochem. J..

[116] Ko S.H., Rho D.J., Jeon J.I., Kim Y.J., Woo H.A., Lee Y.K., Kim J.M. (2016). Bacteroides fragilis Enterotoxin Upregulates Heme Oxygenase-1 in Intestinal Epithelial Cells via a Mitogen-Activated Protein Kinase- and NF-κB-Dependent Pathway, Leading to Modulation of Apoptosis. Infect. Immun..

[117] Purcell R.V., Permain J., Keenan J.I. (2022). Enterotoxigenic Bacteroides fragilis activates IL-8 expression through Stat3 in colorectal cancer cells. Gut Pathog..

[118] Kim J.M., Jung H.Y., Lee J.Y., Youn J., Lee C.H., Kim K.H. (2005). Mitogen-activated protein kinase and activator protein-1 dependent signals are essential for Bacteroides fragilis enterotoxin-induced enteritis. Eur. J. Immunol..

[119] Ye C., Liu X., Liu Z., Pan C., Zhang X., Zhao Z., Sun H. (2024). *Fusobacterium nucleatum* in tumors: From tumorigenesis to tumor metastasis and tumor resistance. Cancer Biol. Ther..

[120] Jeon J.I., Lee K.H., Kim J.M. (2021). Bacteroides fragilis Enterotoxin Upregulates Matrix Metalloproteinase-7 Expression through MAPK and AP-1 Activation in Intestinal Epithelial Cells, Leading to Syndecan-2 Release. Int. J. Mol. Sci..

[121] Lialios P., Alimperti S. (2025). Role of E-cadherin in epithelial barrier dysfunction: Implications for bacterial infection, inflammation, and disease pathogenesis. Front. Cell Infect. Microbiol..

[122] Jasemi S., Molicotti P., Fais M., Cossu I., Simula E.R., Sechi L.A. (2025). Biological Mechanisms of Enterotoxigenic Bacteroides fragilis Toxin: Linking Inflammation, Colorectal Cancer, and Clinical Implications. Toxins.

[123] Hwang S., Gwon S.Y., Kim M.S., Lee S., Rhee K.J. (2013). Bacteroides fragilis Toxin Induces IL-8 Secretion in HT29/C1 Cells through Disruption of E-cadherin Junctions. Immune Netw..

[124] Rêgo A., Araújo-Filho I. (2024). Microbiome and Treatment Resistance in Colorectal Cancer: Mechanisms and Solutions. Biomed. J. Sci. Tech. Res..

[125] Waldner M.J., Foersch S., Neurath M.F. (2012). Interleukin-6—A key regulator of colorectal cancer development. Int. J. Biol. Sci..

[126] Manore S.G., Doheny D.L., Wong G.L., Lo H.W. (2022). IL-6/JAK/STAT3 Signaling in Breast Cancer Metastasis: Biology and Treatment. Front. Oncol..

[127] Song X., Wei C., Li X. (2021). The potential role and status of IL-17 family cytokines in breast cancer. Int. Immunopharmacol..

[128] Benevides L., da Fonseca D.M., Donate P.B., Tiezzi D.G., De Carvalho D.D., de Andrade J.M., Martins G.A., Silva J.S. (2015). IL17 Promotes Mammary Tumor Progression by Changing the Behavior of Tumor Cells and Eliciting Tumorigenic Neutrophils Recruitment. Cancer Res..

[129] Xuan C., Shamonki J.M., Chung A., Dinome M.L., Chung M., Sieling P.A., Lee D.J. (2014). Microbial dysbiosis is associated with human breast cancer. PLoS ONE.

[130] An J., Kwon H., Lim W., Moon B.I. (2022). Staphylococcus aureus-Derived Extracellular Vesicles Enhance the Efficacy of Endocrine Therapy in Breast Cancer Cells. J. Clin. Med..

[131] Bothou A., Zervoudis S., Pappou P., Tsatsaris G., Gerede A., Dragoutsos G., Chalkidou A., Nikolettos K., Tsikouras P. (2022). Mastitis and Risk of Breast Cancer: A Case Control-Retrospective Study and Mini-Review. Maedica.

[132] Chen D., Enroth S., Ivansson E., Gyllensten U. (2014). Pathway analysis of cervical cancer genome-wide association study highlights the MHC region and pathways involved in response to infection. Hum. Mol. Genet.

[133] Mlynarczyk-Bonikowska B., Kowalewski C., Krolak-Ulinska A., Marusza W. (2022). Molecular Mechanisms of Drug Resistance in Staphylococcus aureus. Int. J. Mol. Sci..

[134] Saud Hussein A., Ibraheem Salih N., Hashim Saadoon I. (2021). Effect of Microbiota in the Development of Breast Cancer. Arch. Razi Inst..

[135] Kengne M.F., Mbaveng A.T., Kuete V. (2024). Antibiotic Resistance Profile of Staphylococcus aureus in Cancer Patients at Laquintinie Hospital in Douala, Littoral Region, Cameroon. BioMed Res. Int..

[136] Odunitan T.T., Apanisile B.T., Akinboade M.W., Abdulazeez W.O., Oyaronbi A.O., Ajayi T.M., Oyekola S.A., Ibrahim N.O., Nafiu T., Afolabi H.O. (2024). Microbial mysteries: Staphylococcus aureus and the enigma of carcinogenesis. Microb. Pathog..

[137] Roche F.M., Downer R., Keane F., Speziale P., Park P.W., Foster T.J. (2004). The N-terminal A domain of fibronectin-binding proteins A and B promotes adhesion of Staphylococcus aureus to elastin. J. Biol. Chem..

[138] Pietrocola G., Nobile G., Alfeo M.J., Foster T.J., Geoghegan J.A., De Filippis V., Speziale P. (2019). Fibronectin-binding protein B (FnBPB) from Staphylococcus aureus protects against the antimicrobial activity of histones. J. Biol. Chem..

[139] Badarau A., Trstenjak N., Nagy E. (2017). Structure and Function of the Two-Component Cytotoxins of Staphylococcus aureus—Learnings for Designing Novel Therapeutics. Adv. Exp. Med. Biol..

[140] Kaneko J., Kamio Y. (2004). Bacterial two-component and hetero-heptameric pore-forming cytolytic toxins: Structures, pore-forming mechanism, and organization of the genes. Biosci. Biotechnol. Biochem..

[141] Alonzo F., Torres V.J. (2014). The bicomponent pore-forming leucocidins of Staphylococcus aureus. Microbiol. Mol. Biol. Rev..

[142] Wei Y., Sandhu E., Yang X., Yang J., Ren Y., Gao X. (2022). Bidirectional Functional Effects of Staphylococcus on Carcinogenesis. Microorganisms.

[143] Chambers H.F., Deleo F.R. (2009). Waves of resistance: Staphylococcus aureus in the antibiotic era. Nat. Rev. Microbiol..

[144] Lowy F.D. (1998). Staphylococcus aureus infections. N. Engl. J. Med..

[145] David M.Z., Daum R.S. (2010). Community-associated methicillin-resistant Staphylococcus aureus: Epidemiology and clinical consequences of an emerging epidemic. Clin. Microbiol. Rev..

[146] Tzeng A., Sangwan N., Jia M., Liu C.C., Keslar K.S., Downs-Kelly E., Fairchild R.L., Al-Hilli Z., Grobmyer S.R., Eng C. (2021). Human breast microbiome correlates with prognostic features and immunological signatures in breast cancer. Genome Med..

[147] Klann E., Williamson J.M., Tagliamonte M.S., Ukhanova M., Asirvatham J.R., Chim H., Yaghjyan L., Mai V. (2020). Microbiota composition in bilateral healthy breast tissue and breast tumors. Cancer Causes Control.

[148] Urbaniak C., Cummins J., Brackstone M., Macklaim J.M., Gloor G.B., Baban C.K., Scott L., O’Hanlon D.M., Burton J.P., Francis K.P. (2014). Microbiota of human breast tissue. Appl. Environ. Microbiol..

[149] Herrera-Quintana L., Vázquez-Lorente H., Lopez-Garzon M., Cortés-Martín A., Plaza-Diaz J. (2024). Cancer and the Microbiome of the Human Body. Nutrients.

[150] Cheung G.Y.C., Bae J.S., Otto M. (2021). Pathogenicity and virulence of Staphylococcus aureus. Virulence.

[151] Speziale P., Pietrocola G. (2021). Staphylococcus aureus induces neutrophil extracellular traps (NETs) and neutralizes their bactericidal potential. Comput. Struct. Biotechnol. J..

[152] Coffelt S.B., Kersten K., Doornebal C.W., Weiden J., Vrijland K., Hau C.S., Verstegen N.J.M., Ciampricotti M., Hawinkels L., Jonkers J. (2015). IL-17-producing γδ T cells and neutrophils conspire to promote breast cancer metastasis. Nature.

[153] Akula S., Gonzalez C.G., Kermet S., Burleson M. (2024). Natural compounds solasonine and alisol B23-acetate target GLI3 signaling to block oncogenesis in MED12-altered breast cancer. Mol. Biol. Res. Commun..

[154] Bhole R., Shinkar J., Labhade S., Karwa P., Kapare H. (2025). MED12 dysregulation: Insights into cancer and therapeutic resistance. Naunyn-Schmiedeberg’s Arch. Pharmacol..

[155] Cossu C., Di Lorenzo A., Fiorilla I., Todesco A.M., Audrito V., Conti L. (2023). The Role of the Toll-like Receptor 2 and the cGAS-STING Pathways in Breast Cancer: Friends or Foes?. Int. J. Mol. Sci..

[156] Chandrasekar S.A., Palaniyandi T., Parthasarathy U., Surendran H., Viswanathan S., Wahab M.R.A., Baskar G., Natarajan S., Ranjan K. (2023). Implications of Toll-like receptors (TLRs) and their signaling mechanisms in human cancers. Pathol. Res. Pract..

[157] Yao Y., Li X., Xu B., Luo L., Guo Q., Wang X., Sun L., Zhang Z., Li P. (2022). Cholecystectomy promotes colon carcinogenesis by activating the Wnt signaling pathway by increasing the deoxycholic acid level. Cell Commun. Signal..

[158] Bodai B.I., Nakata T.E. (2020). Breast Cancer: Lifestyle, the Human Gut Microbiota/Microbiome, and Survivorship. Perm. J..

[159] Chen K.L., Madak-Erdogan Z. (2016). Estrogen and Microbiota Crosstalk: Should We Pay Attention?. Trends Endocrinol. Metab..

[160] Alves C.L., Ditzel H.J. (2023). Drugging the PI3K/AKT/mTOR Pathway in ER+ Breast Cancer. Int. J. Mol. Sci..

[161] Gido C.D., Herdendorf T.J., Geisbrecht B.V. (2023). Characterization of two distinct neutrophil serine protease-binding modes within a Staphylococcus aureus innate immune evasion protein family. J. Biol. Chem..

[162] Goedert J.J., Jones G., Hua X., Xu X., Yu G., Flores R., Falk R.T., Gail M.H., Shi J., Ravel J. (2015). Investigation of the association between the fecal microbiota and breast cancer in postmenopausal women: A population-based case-control pilot study. J. Natl. Cancer Inst..

[163] Byrd A.L., Liu M., Fujimura K.E., Lyalina S., Nagarkar D.R., Charbit B., Bergstedt J., Patin E., Harrison O.J., Quintana-Murci L. (2021). Gut microbiome stability and dynamics in healthy donors and patients with non-gastrointestinal cancers. J. Exp. Med..

[164] Plottel C.S., Blaser M.J. (2011). Microbiome and malignancy. Cell Host Microbe.

[165] Yoshimoto S., Loo T.M., Atarashi K., Kanda H., Sato S., Oyadomari S., Iwakura Y., Oshima K., Morita H., Hattori M. (2013). Obesity-induced gut microbial metabolite promotes liver cancer through senescence secretome. Nature.

[166] Buffie C.G., Pamer E.G. (2013). Microbiota-mediated colonization resistance against intestinal pathogens. Nat. Rev. Immunol..

[167] Kornbluth A.A., Danzig J.B., Bernstein L.H. (1989). Clostridium septicum infection and associated malignancy. Report of 2 cases and review of the literature. Medicine.

[168] Albenberg L.G., Wu G.D. (2014). Diet and the intestinal microbiome: Associations, functions, and implications for health and disease. Gastroenterology.

[169] Guo P., Zhang K., Ma X., He P. (2020). Clostridium species as probiotics: Potentials and challenges. J. Anim. Sci. Biotechnol..

[170] Riviere A., Selak M., Lantin D., Leroy F., De Vuyst L. (2016). Bifidobacteria and Butyrate-Producing Colon Bacteria: Importance and Strategies for Their Stimulation in the Human Gut. Front. Microbiol..

[171] Umesaki Y., Setoyama H., Matsumoto S., Imaoka A., Itoh K. (1999). Differential Roles of Segmented Filamentous Bacteria and Clostridia in Development of the Intestinal Immune System. Infect. Immun..

[172] Buffie C.G., Bucci V., Stein R.R., McKenney P.T., Ling L., Gobourne A., No D., Liu H., Kinnebrew M., Viale A. (2015). Precision microbiome reconstitution restores bile acid mediated resistance to Clostridium difficile. Nature.

[173] Gerard P. (2013). Metabolism of cholesterol and bile acids by the gut microbiota. Pathogens.

[174] Dickert S., Pierik A.J., Buckel W. (2002). Molecular characterization of phenyllactate dehydratase and its initiator from Clostridium sporogenes. Mol. Microbiol..

[175] Bose C., Uczkowski N.G., Sukla K., Raval N., Haque M.M., Zhang Y., Varma B., Satagopan J.M. (2025). Breast cancer and microbiome: A systematic review highlighting challenges for clinical translation. BMC Women’s Health.

[176] Moreira-Pinto J., Passos-Coelho J.L., Lopes F., Ataide M., Oliveira P. (2020). Fatal Clostridium septicum febrile neutropenia during adjuvant chemotherapy for early breast cancer. BMJ Case Rep..

[177] Holub M., Řezáč D., Čurdová M. (2021). Fatal Neutropenic Colitis and Clostridium Septicum Bacteremia in a Breast Cancer Patient. Prague Med. Rep..

[178] Zengul A.G., Demark-Wahnefried W., Barnes S., Morrow C.D., Bertrand B., Berryhill T.F., Fruge A.D. (2021). Associations between Dietary Fiber, the Fecal Microbiota and Estrogen Metabolism in Postmenopausal Women with Breast Cancer. Nutr. Cancer.

[179] Murray W.R., Blackwood A., Calman K.C., MacKay C. (1980). Faecal bile acids and clostridia in patients with breast cancer. Br. J. Cancer.

[180] Hieken T.J., Chen J., Chen B., Johnson S., Hoskin T.L., Degnim A.C., Walther-Antonio M.R., Chia N. (2022). The breast tissue microbiome, stroma, immune cells and breast cancer. Neoplasia.

[181] Chiang J.Y. (2013). Bile acid metabolism and signaling. Compr. Physiol..

[182] Nagathihalli N.S., Beesetty Y., Lee W., Washington M.K., Chen X., Lockhart A.C., Merchant N.B. (2014). Novel mechanistic insights into ectodomain shedding of EGFR Ligands Amphiregulin and TGF-alpha: Impact on gastrointestinal cancers driven by secondary bile acids. Cancer Res..

[183] Wang L., Gong Z., Zhang X., Zhu F., Liu Y., Jin C., Du X., Xu C., Chen Y., Cai W. (2020). Gut microbial bile acid metabolite skews macrophage polarization and contributes to high-fat diet-induced colonic inflammation. Gut Microbes.

[184] Fierer N., Leung P.M., Lappan R., Eisenhofer R., Ricci F., Holland S.I., Dragone N., Blackall L.L., Dong X., Dorador C. (2025). Guidelines for preventing and reporting contamination in low-biomass microbiome studies. Nat. Microbiol..

[185] Salter S.J., Cox M.J., Turek E.M., Calus S.T., Cookson W.O., Moffatt M.F., Turner P., Parkhill J., Loman N.J., Walker A.W. (2014). Reagent and laboratory contamination can critically impact sequence-based microbiome analyses. BMC Biol..

[186] Eisenhofer R., Minich J.J., Marotz C., Cooper A., Knight R., Weyrich L.S. (2019). Contamination in Low Microbial Biomass Microbiome Studies: Issues and Recommendations. Trends Microbiol..

[187] Pollock J., Glendinning L., Wisedchanwet T., Watson M. (2018). The Madness of Microbiome: Attempting to Find Consensus “Best Practice” for 16S Microbiome Studies. Appl. Environ. Microbiol..

[188] Knight R., Vrbanac A., Taylor B.C., Aksenov A., Callewaert C., Debelius J., Gonzalez A., Kosciolek T., McCall L.I., McDonald D. (2018). Best practices for analysing microbiomes. Nat. Rev. Microbiol..

[189] Dethlefsen L., Relman D.A. (2011). Incomplete recovery and individualized responses of the human distal gut microbiota to repeated antibiotic perturbation. Proc. Natl. Acad. Sci. USA.

[190] Jernberg C., Löfmark S., Edlund C., Jansson J.K. (2007). Long-term ecological impacts of antibiotic administration on the human intestinal microbiota. ISME J..

[191] Routy B., Le Chatelier E., Derosa L., Duong C.P.M., Alou M.T., Daillère R., Fluckiger A., Messaoudene M., Rauber C., Roberti M.P. (2018). Gut microbiome influences efficacy of PD-1-based immunotherapy against epithelial tumors. Science.

[192] Derosa L., Hellmann M.D., Spaziano M., Halpenny D., Fidelle M., Rizvi H., Long N., Plodkowski A.J., Arbour K.C., Chaft J.E. (2018). Negative association of antibiotics on clinical activity of immune checkpoint inhibitors in patients with advanced renal cell and non-small-cell lung cancer. Ann. Oncol..

